# Comparison of the FASD 4-Digit Code and Hoyme et al. 2016 FASD diagnostic guidelines

**DOI:** 10.12715/apr.2017.4.13

**Published:** 2017-10-30

**Authors:** Susan J. Astley, Julia M. Bledsoe, Julian K. Davies, John C. Thorne

**Affiliations:** 1 University of Washington, Seattle, WA, USA

## Abstract

**Background:**

As clinicians strive to achieve consensus worldwide on how best to diagnose fetal alcohol spectrum disorders (FASD), the most recent FASD diagnosstic systems exhibit convergence and divergence. Applying these systems to a single clinical population illustrates contrasts between them, but validation studies are ultimately required to identify the best system. Currently, only the 4-Digit Code has published comprehensive validation studies.

**Methods:**

The 4-Digit Code and Hoyme 2016 FASD systems were applied to the records of 1,392 patients evaluated for FASD at the University of Washington to: 1) Compare the diagnostic criteria and tools used by each system, 2) Compare the prevalence and concordance of diagnostic outcomes and assess measures of validity.

**Results:**

Only 38% of patients received concordant diagnoses. The Hoyme criteria rendered half as many diagnoses under the umbrella of FASD (n=558) as the 4-Digit Code (n=1,092) and diagnosed a much higher proportion (53%) as fetal alcohol syndrome/partial fetal alcohol syndrome (FAS/PFAS) than the 4-Digit Code (7%). Key Hoyme factors contributing to discordance included relaxation of facial criteria (40% had the Hoyme FAS face, including patients with confirmed absence of alcohol exposure); setting alcohol exposure thresholds prevented 1/3 with confirmed exposure from receiving FAS/FASD diagnoses; and setting minimum age limits for Alcohol-Related Neurodevelopmental Disorder prevented 79% of alcohol-exposed infants with neurodevelopmental impairment a FASD diagnosis. The Hoyme Lip/Philtrum Guides differ substantively from the 4-Digit Lip-Philtrum Guides and thus are not valid for use with the 4-Digit Code.

**Conclusions:**

All FASD diagnostic systems need to publish comprehensive validation studies to identify which is the most accurate, reproducible, and medically valid.

## Introduction

As the field of fetal alcohol spectrum disorders (FASD) strives to achieve consensus worldwide on how best to diagnose FASD, the most recent versions of published guidelines (4-Digit Code, 2004 [1]) Canadian, 2015 [2], Hoyme et al., 2016 [3], and Australian, 2016 [4]) show both convergence and divergence. The new Canadian and Australian systems share many features in common, but diverge substantially from the 4-Digit Code and Hoyme et al. systems by dropping the growth deficiency criteria [5] and adopting a nomenclature (FASD with the face, and FASD without the face) that no longer reflects the spectrum of outcome. The 4-Digit Code [1] and Hoyme et al. [3] criteria continue to generate a spectrum of diagnoses under the umbrella of FASD (fetal alcohol syndrome (FAS), partial FAS (PFAS), Alcohol Related Neurodevelopmental Disorder (ARND), Static Encephalopathy/Alcohol Exposed (SE/AE), Neurobehavioral Disorder/Alcohol Exposed (ND/AE), and Alcohol Related Birth Defects (ARBD)) and maintain the 3 original core diagnostic criteria (growth deficiency, facial anomalies, and CNS abnormalities). The 4-Digit Code and Hoyme et al. systems differ in their diagnostic nomenclature, diagnostic tools, and the specific criteria used to generate each diagnosis. Comparing the diagnostic outcomes generated by the different systems when applied to a single clinical population serves to illustrate the major contrasts and similarities between the systems, but empirical validation studies are ultimately needed to identify the best system.

The objectives of this study were to:

1. Compare the tools and criteria used by the 4-Digit Code and Hoyme et al. 2016 FASD diagnostic systems.
2. Administer each system to the records of 1,392 patients to:
  - Compare the prevalence of FASD diagnoses produced by each system.
  - Assess diagnostic concordance between the two systems.
  - Compare measures of validity applied to each system.

The outcomes of Objective 1 helped guide the study design (methods and study population) for Objective 2. Thus, the methods and results for Objective 1 are presented first, followed by the methods and results for Objective 2.

## Objective 1. Comparison of the diagnostic tools and Criteria

### Methods

#### Tools

##### Lip-philtrum guides:

**Figure 1.**
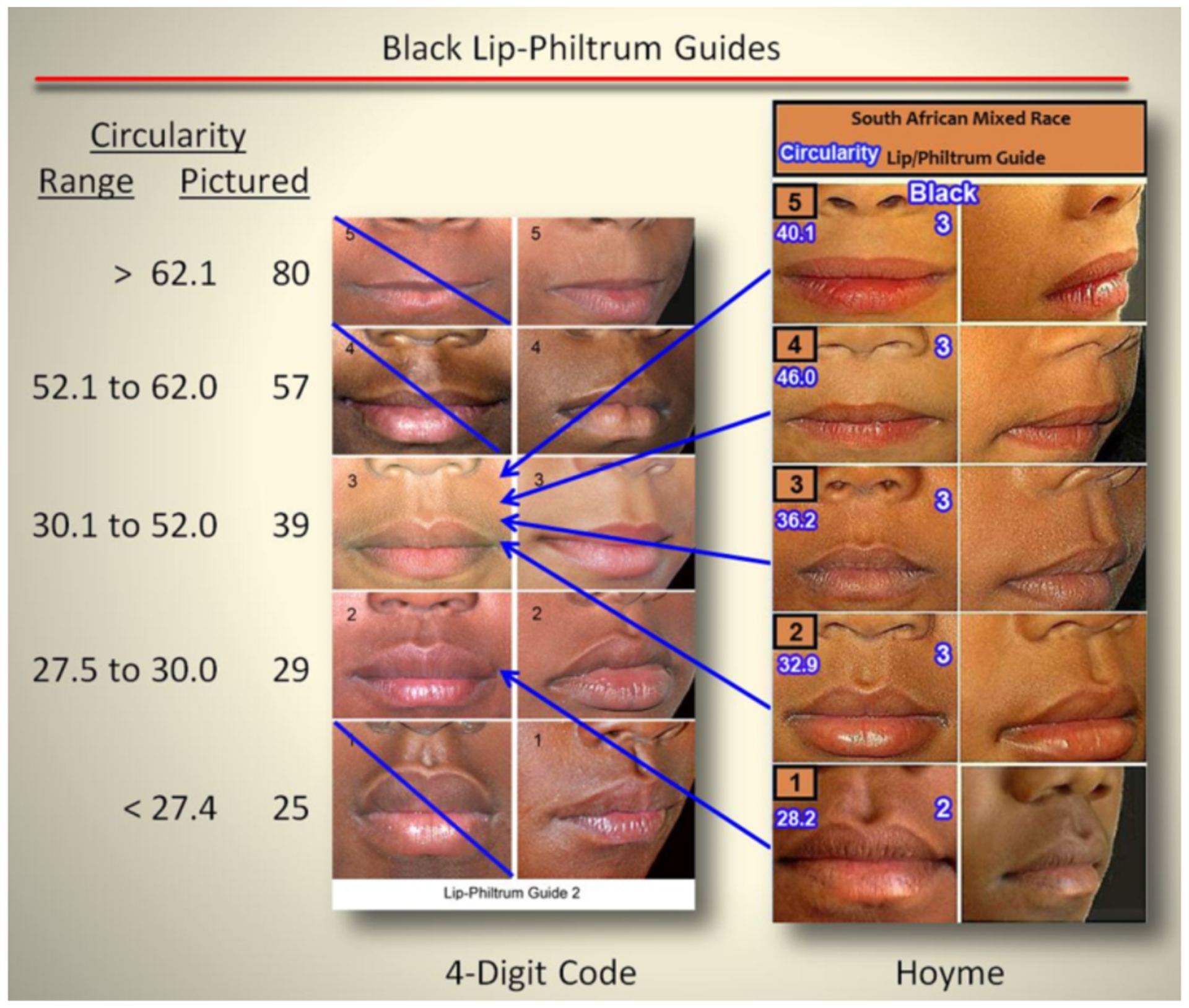
The Hoyme South-African Mixed-Race Lip/Philtrum Guide differs from the “African-American” 4-Digit Code Lip-Philtrum Guide 2 *Upper lip*: The objective measure of upper lip thinness (circularity = perimeter^2^/area) is printed to the left of the 4-Digit Code Guide and in white font with a blue border on the Hoyme Guide. For example, the 4-Digit Code Rank 4 lip has a circularity of 57 and is defined by the range of circularities 52.1 to 62.0. The Hoyme Rank 4 lip has a circularity of 46.0, thus is equivalent to the 4-Digit Code Rank 3 lip. Circularity confirmed the Hoyme et al. Ranks 1, 2, 3, 4 and 5 lips were equivalent to the 4-Digit Ranks 2, 3, 3, 3, and 3 respectively. There are no lip images on the Hoyme et al. Guide that correspond to (fall within the circularity ranges that define) the 4-Digit Ranks 1, 4, or 5. Lips on the Hoyme et al. Guide do not increase in thinness in linear fashion as they do on the 4-Digit Code Guide 2. The Hoyme et al. Rank 5 lip is thicker (circularity 40.1) than the Hoyme et al. Rank 4 lip (circularity 46.0). Most importantly, the Hoyme et al. Rank 4 lip (the clinical cut-off for FAS) is thicker than the 4-Digit Code Rank 4 lip. The Hoyme et al. Rank 4 lip (circularity 46.0) falls within the circularity range depicted by the 4-Digit Rank 3 lip (30.1 to 52.0). The Hoyme et al. Rank 5 lip (circularity 40.1) is substantially thicker than the 4-Digit Rank 5 lip (circularity 80). *Philtrum*: The Rank 1 through 5 philtrums depicted on both guides appeared broadly equivalent by visual inspection. Based on our findings and the findings of Hoyme et al. [6], the South African Mixed Race Lip/Philtrum Guide is not appropriate for use on an African American population. (South African Mixed Race Lip/Philtrum Guide used with permission from Wiley & Sons, Inc).

**Figure 2.**
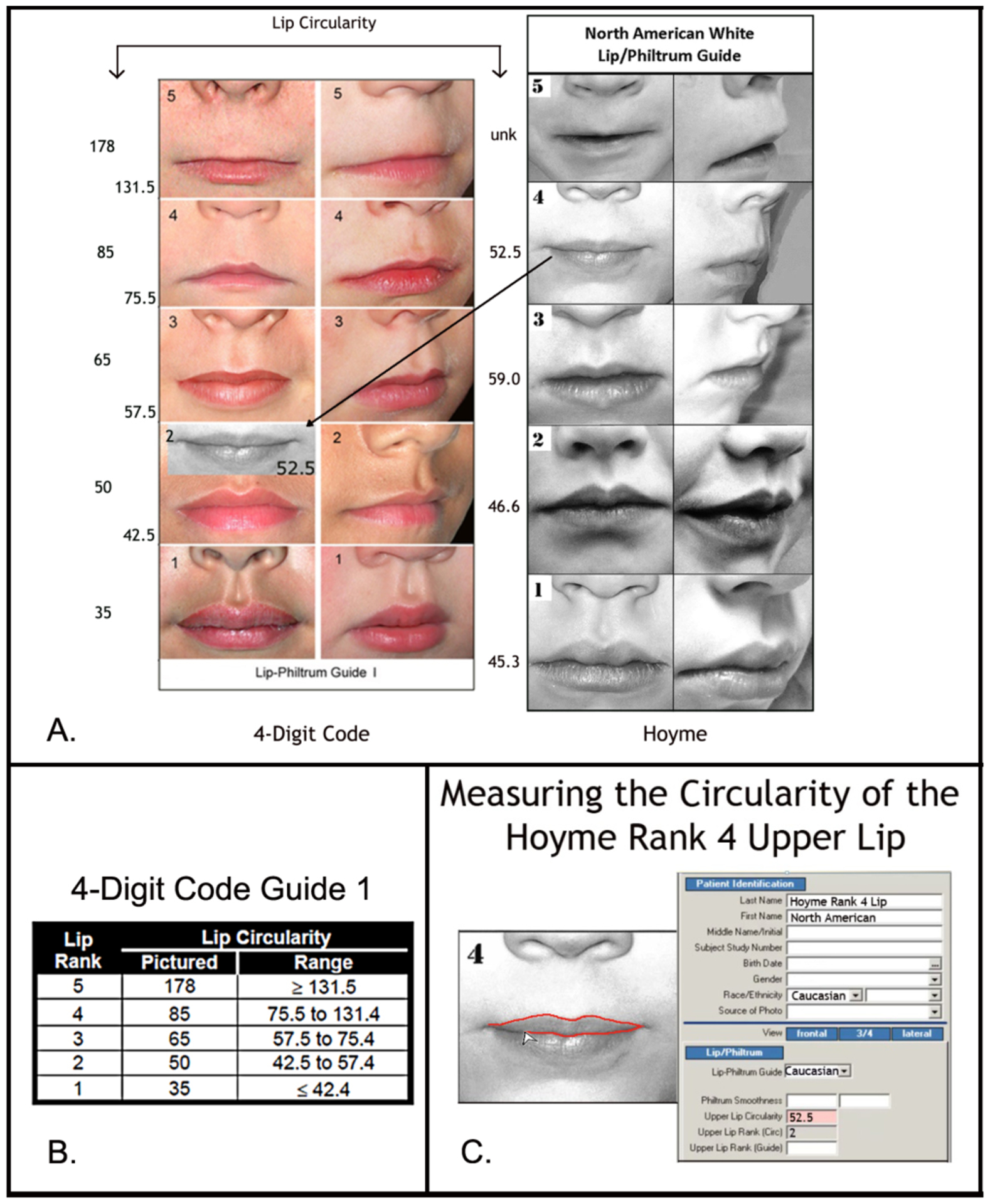
The Hoyme North-American White Lip/Philtrum Guide differs from the 4-Digit Code “Caucasian” Lip-Philtrum Guide 1 The Rank 1 through 5 philtrums depicted on both Guides appear broadly equivalent, but the upper lips are substantially different. A) Lip circularity (perimeter^2^/area) is printed to the left of each guide. B) The range of circularities that define each 4-Digit Code Lip Rank are presented in the Lip Circularity table printed on the backside of the 4-Digit Code Lip-Philtrum Guide. C) The FAS Facial Photographic Analysis Software [7] computes circularity when the User outlines the vermilion border of the upper lip (click on video link for demonstration http://depts.washington.edu/fasdpn/movie/Fig2Cvideo.mp4). Lip circularity confirms that the Hoyme et al. Rank 1, 2, 3, 4 and 5 upper lips are equivalent to the 4-Digit Code Ranks 2, 2, 3, 2 and 5 respectively. There is no lip image on the Hoyme et al. Guide that reflects the 4-Digit Rank 1 or Rank 4 lips. The lips on the 4-Digit Guide become progressively thinner (circularity becomes progressively larger) with increasing Rank. This is not true for the Hoyme et al. Guide. The circularity of the Hoyme et al. Rank 4 lip (the clinical cut-off for FAS) is 52.5, confirming it falls within the circularity range of the 4-Digit Code Rank 2 lip. The black and white overlay (A) of the Hoyme et al. Rank 4 lip on the 4-Digit Code Guide 1 demonstrates both visually and numerically that the Hoyme et al. Rank 4 lip is substantially thicker than the 4-Digit Code Rank 4 lip. This analysis confirms the Hoyme et al. North American White Lip/Philtrum Guide is not a valid tool for use with the FASD 4-Digit Diagnostic Code.(North American White Lip/Philtrum Guide used with permission from the American Academy of Pediatrics).

Both diagnostic systems provide 5-point, pictorial lip-philtrum guides for ranking the magnitude of philtrum smoothness and upper lip thinness. The 4-Digit Code provides two guides: Lip-Philtrum Guide 1 for Caucasians and all races with thinner upper lips like Caucasian, and Lip-Philtrum Guide 2 for African Americans and all races with thicker upper lips like African Americans (Figures 1 and 2). Hoyme et al. have also introduced two lip/philtrum guides: the North American Lip/Philtrum Guide [3] produced from a U.S. white population and the South African Mixed Race Lip/Philtrum Guide [6] produced from a Cape Coloured (mixed race) population in the Western Cape Province (Figures 1 and 2).

- *Philtrum:* The Rank 1–5 philtrums depicted on the 4-Digit Code Caucasian and Hoyme et al. North American guides were visually compared to determine if the magnitude of philtrum depth or smoothness depicted by each Rank was comparable between the two guides (e.g. was the Rank 4 philtrum smoothness depicted on the 4-Digit Guide the same as the Rank 4 philtrum smoothness depicted on the Hoyme et al. Guide?). This visual comparison was repeated for the 4-Digit African American and Hoyme et al. South African guides.
- *Upper lip:* The Rank 1–5 lips depicted on the 4-Digit Code Caucasian and Hoyme et al. North American guides were compared using the objective, quantitative measure of lip thinness called lip circularity (perimeter2/area) generated by the FAS Facial Photographic Analysis Software [7]. Circularity is computed by outlining the vermilion border of the upper lip with the mouse (Figure 2C); the thinner the lip, the bigger the circularity. Lip Ranks 1–5 on the 4-Digit Code Lip-Philtrum Guides are case-defined by the range of lip circularities posted on the backside of each Lip-Philtrum Guide (Figure 2B). Lip circularity was computed for each lip on the Hoyme et al. North American Lip/Philtrum Guide. The lip circularities on the 4-Digit Code Caucasian and Hoyme et al. North American guides were compared to determine if the magnitude of lip thinness depicted by each Rank was comparable (e.g., was the Rank 4 lip thinness depicted on the 4-Digit Caucasian Guide the same as the Rank 4 lip thinness depicted on the Hoyme et al. North American Guide?). This comparison of lip circularities was repeated for the 4-Digit Code African American and Hoyme et al. South African guides

##### Facial analysis software:

The 4-Digit Code advises measuring the facial features from 2D digital photos using the FAS Facial Photographic Analysis Software [7]. The authors of the Hoyme et al. system “feel direct examinations of facial features are more practical in an office setting.” Since empirical studies have already confirmed the superior accuracy of the photo versus direct method of facial measurement [8, 9], a formal assessment of photo versus direct measurement of facial features was not repeated in this study.

#### Diagnostic nomenclature and criteria

Figures were created to illustrate the key contrasts between the diagnoses generated by each system, the nomenclature assigned to each diagnosis, and the diagnostic criteria.

### Results

#### Tools: Contrasts in lip-philtrum guides

The Hoyme et al 2015 South African Mixed Race Lip/Philtrum Guide [6] does not match the “African American” 4-Digit Code Lip-Philtrum Guide 2 (Figure 1).

- *Philtrum:* The Rank 1 through 5 philtrums depicted on both guides appeared broadly equivalent by visual inspection.
- *Upper lip:* Circularity confirms the Hoyme et al. Rank 4 lip (the clinical cut-off for FAS) is thicker than the 4-Digit Code Rank 4 lip (e.g., it is equivalent to the 4-Digit Code Rank 3 lip). Unlike the 4-Digit Code Lip-Philtrum Guide, the lips pictured on the Hoyme et al. Guide do not progressively become thinner as Rank increases and no lip on the Hoyme et al. Guide is equivalent to the 4-Digit Code Rank 1, 4 or 5 lips. Based on our findings here and the findings of Hoyme et al. [6], the South African Mixed Race Lip/Philtrum Guide is not appropriate for use on an African American population and thus was not used to address Study Objective 2. The study population for Objective 2 was adjusted accordingly (as described below) to accommodate this finding.

The Hoyme et al. 2016 [3] North American White Lip/Philtrum Guide does not match the “Caucasian” 4-Digit Code Lip-Philtrum Guide 1 (Figure 2).

- *Philtrum:* The Rank 1 through 5 philtrums depicted on both guides appeared broadly equivalent by visual inspection.
- *Upper lip:* Circularity (perimeter^2^/area) confirms the Hoyme et al. Rank 4 lip (the clinical cut-off for FAS) is substantially thicker than the 4-Digit Code Rank 4 lip (e.g., it is equivalent to the 4-Digit Code Rank 2 lip). The 4-Digit Code defines the Rank 2 lip as within the normal range, slightly thicker than the population mean depicted by Lip Rank 3. Unlike the 4-Digit Code Lip-Philtrum Guide 1, the lips pictured on the Hoyme et al. Guide do not progressively become thinner as Rank increases and no lip on the Hoyme et al. Guide is equivalent to the 4-Digit Code Rank 1 or Rank 4 lips.

Despite the contrasts between the two lip/philtrum guides, both are intended for use on North American Caucasian populations and thus were used to address Objective 2 below.

#### Contrasts in diagnoses and nomenclature

**Figure 3.**
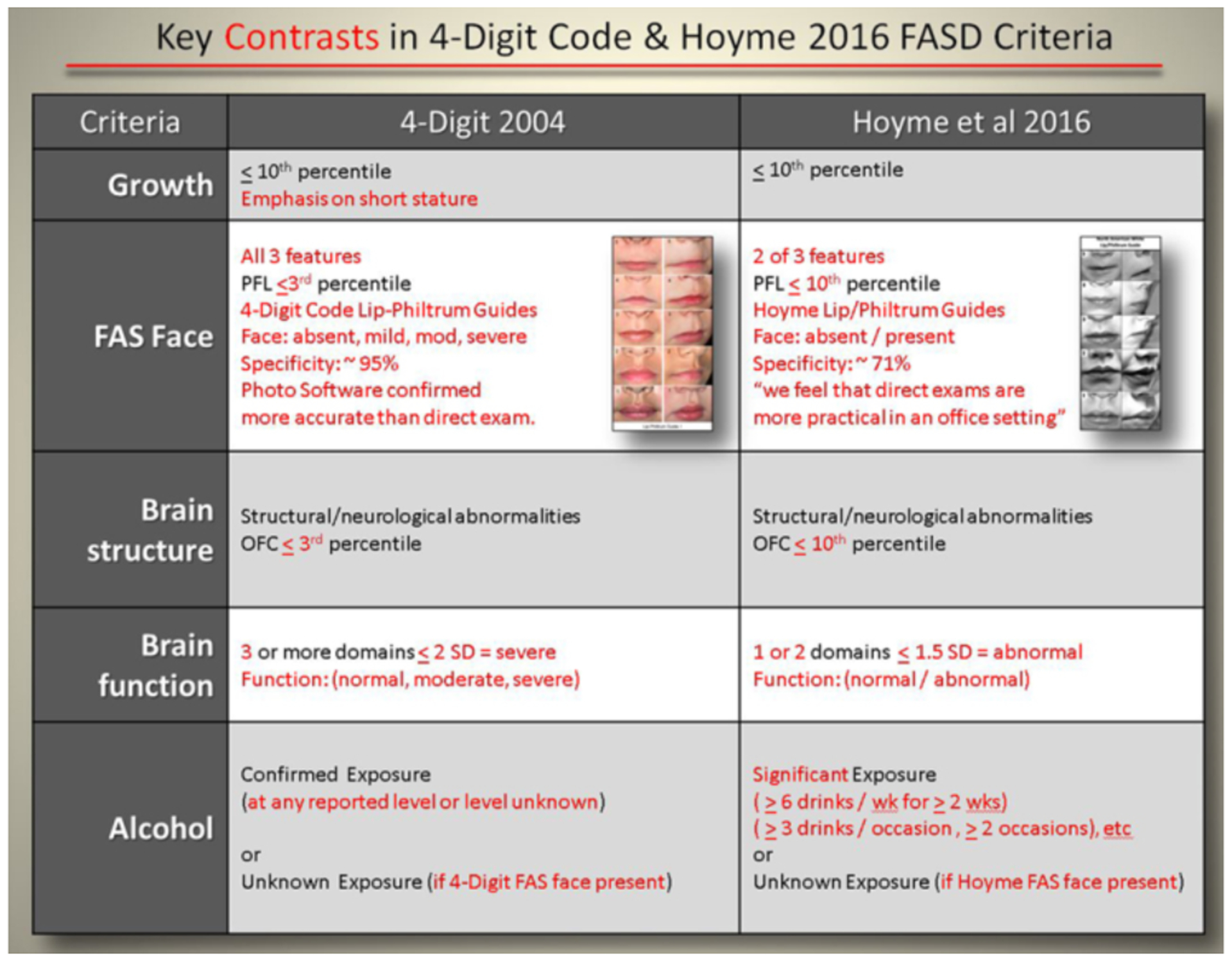
Key contrasts between the 4-Digit Code and Hoyme et al. 2016 FASD diagnostic criteria Key contrasts between the two diagnostic systems are presented in red font. Full criteria are presented in the 4-Digit Code [1] and Hoyme et al. [3] published guidelines.

**Figure 4.**
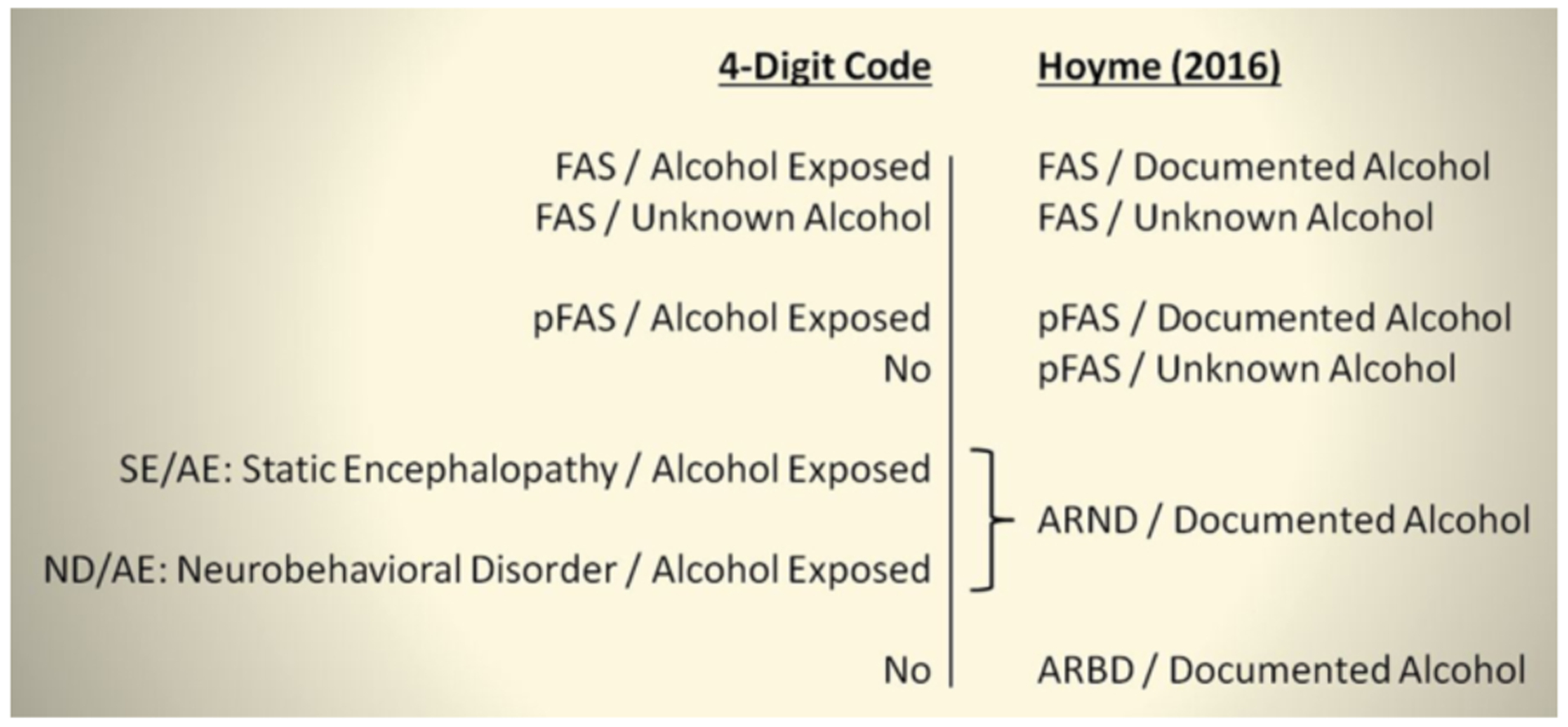
Key contrasts between the 4-Digit Code and Hoyme FASD diagnostic nomenclature The 4-Digit Code [1] defines five FASD diagnostic categories, the Hoyme et al. [3} defines six. Although both diagnostic systems have three diagnostic categories that share the same name, the criteria used to define each are markedly different between the two systems. The 4-Digit Code does not include diagnostic categories labeled pFAS/Unknown Alcohol or ARBD. The diagnosis SE/AE is defined by severe structural and/or functional CNS abnormalities, but lacks the physical characteristics that would qualify for FAS or pFAS. ND/AE is defined by moderate functional CNS abnormalities. The CNS functional abnormalities of ARND are broadly equivalent to the moderate and severe CNS functional abnormalities defined by ND/AE and SE/AE combined. The 4-Digit Code Diagnostic Categories that case-define each diagnosis [1] are as follows: FAS/Alcohol Exposed (Category A); FAS/Unknown Alcohol (B); PFAS/Alcohol Exposed (C); SE/AE (E,F); and ND/AE (G,H). ARND: Alcohol Related Neurodevelopmental Disorder; ARBD: Alcohol Related Birth Defects; FAS: fetal alcohol syndrome; FASD: fetal alcohol spectrum disorders; ND/AE: neurobehavioral disorder/alcohol exposed; pFAS: partial fetal alcohol syndrome; SE/AE: static encephalopathy/alcohol exposed.

The key contrasts between the 4-Digit Code and Hoyme et al diagnoses and nomenclature are highlighted in Figures 3 and 4, respectively.

#### Contrasts in diagnostic criteria

##### Growth deficiency:

The Hoyme et al criteria use the same cut-off (prenatal or postnatal height and/or weight ≤ 10th percentile) to define growth deficiency as the 4-Digit Code, but the Hoyme et al. criteria classify growth deficiency on a dichotomous scale (present/absent), whereas the 4-Digit Code ranks growth deficiency on a 4-point ordinal scale with emphasis on short stature; a method that is confirmed to be highly predictive of CNS dysfunction [5].

##### Facial phenotype:

**Figure 5.**
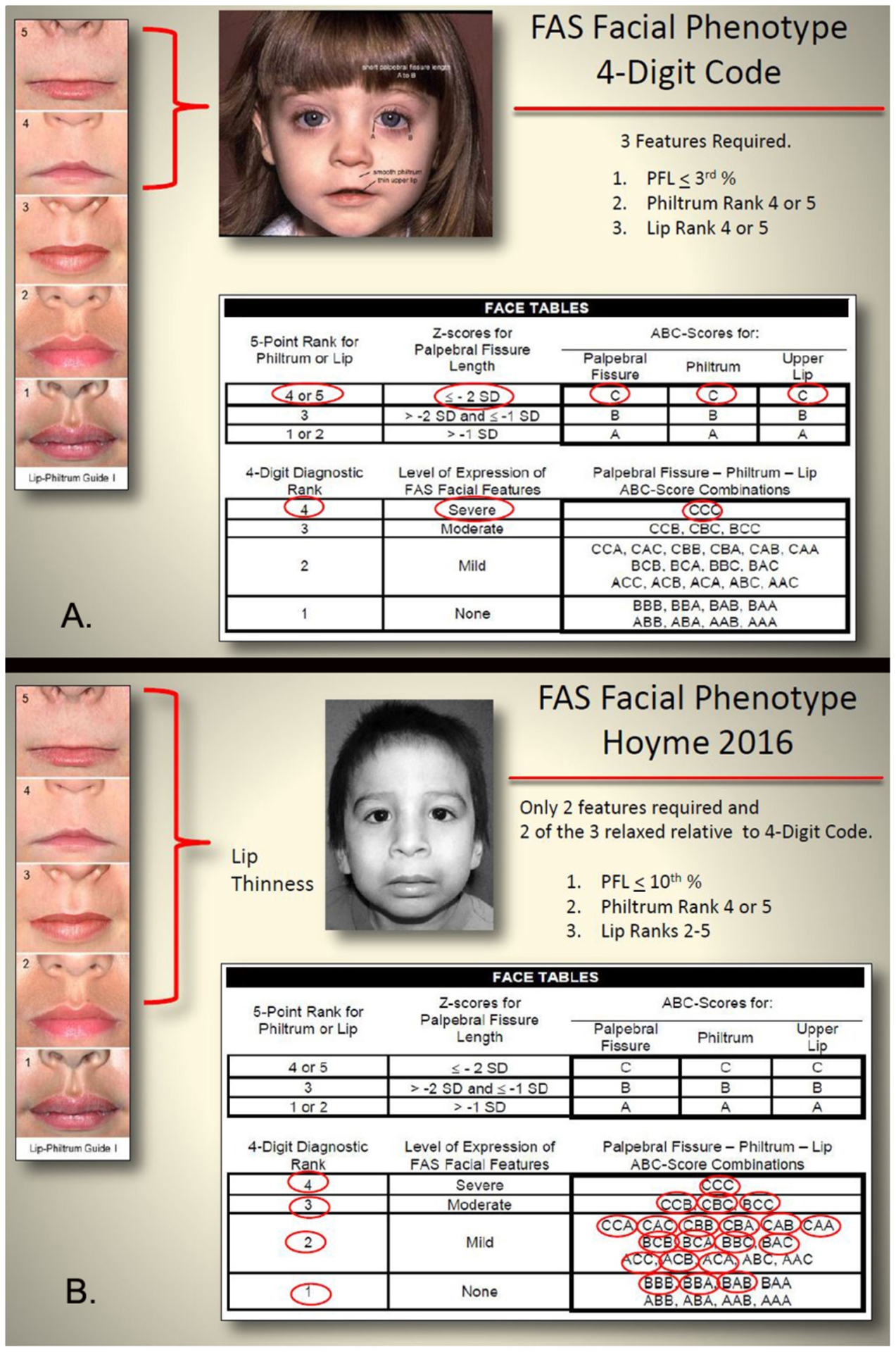
The Hoyme FAS face is substantially relaxed relative to the 4-Digit Code FAS face. A) The 4-Digit Code uses the Facial ABC-Score to document all combinations of expression of the 3 FAS facial features. These ABC-Scores are clustered to define the 4-Digit Code Face Ranks 1–4. The 4-Digit Code FAS facial phenotype is defined by a single ABC-Score (Facial ABC-Score CCC, Face Rank 4). **B)** In contrast, the relaxation of the Hoyme et al. FAS facial criteria (e.g., only 2 of 3 features required, PFL relaxed to 10^th^ percentile, and Rank 4 lip relaxed to 4-Digit Rank 2 lip) results in most every 4-Digit Code Facial ABC-Score meeting the relaxed criteria, including ABC-Scores that define Face Ranks 1 and 2. Clinically, the 4-Digit Code classifies Rank 1 and 2 facial phenotypes as being within the normal range. (FAS facial phenotype from Hoyme et al, 2016 used with permission from the American Academy of Pediatrics).

When compared to the 4-Digit Code Rank 4 FAS facial phenotype, the Hoyme et al. FAS facial phenotype is substantially relaxed. This is best illustrated using the 4-Digit Code Facial ABC-Score printed on the backside of the 4-Digit Code “Caucasian” Lip-Philtrum Guide 1 (Figure 5). The 4-Digit Code FAS facial phenotype is defined by a single ABC-Score (Facial ABC-Score CCC, Face Rank 4) (Figure 5A). The three letters “CCC” reflect the magnitude of expression of the short palpebral fissure length (PFL), smooth philtrum, and thin upper lip in that order. C reflects severe expression in the FAS range, B reflects moderate expression, and A reflects normal expression. The Hoyme et al. FAS facial criteria are relaxed relative to the 4-Digit Code in three ways:

1. Only 2 of 3 cardinal features are required.
2. The PFL is relaxed to the 10th percentile.
3. As shown in our analysis above, the Rank 4 lip on the Hoyme et al. North American Lip/Philtrum Guide has a circularity equivalent to the Rank 2 lip on the 4-Digit Lip-Philtrum Guide 1.

**Figure 6.**
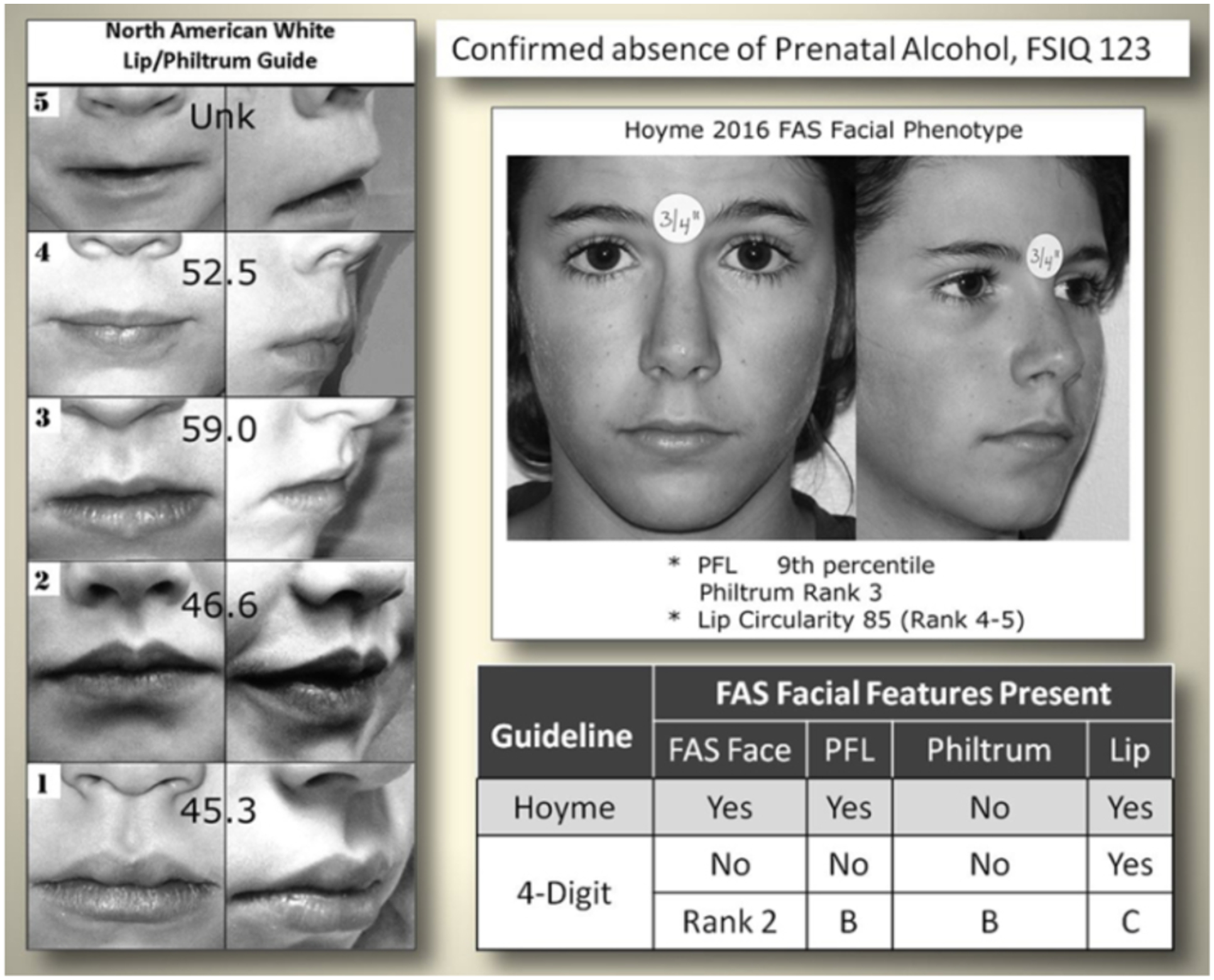
The Hoyme FAS facial criteria are so relaxed even individuals with confirmed absence of prenatal alcohol exposure meet the criteria. When the Hoyme et al. [3] FAS facial criteria were applied to an adolescent with high function (FSIQ 123) and confirmed absence of prenatal alcohol exposure, the adolescent met the Hoyme et al. criteria for the full FAS facial phenotype. When measured directly and with the FAS Facial Photographic Analysis Software, she presented with 2 of the 3 features: palpebral fissure length (PFL) ≤10th percentile using the Stromland Caucasian PFL charts and a thin upper lip ≥ Rank 4 on the Hoyme North American Lip/Philtrum Guide. Both visual inspection and lip circularity (85) confirm her lip was as thin or thinner than the Hoyme Rank 4 lip (circularity 52.5). In contrast, the 4-Digit Code FAS facial criteria classified her within the normal range (Face ABC-Score BBC, Face Rank 2), presenting with just 1 of the 3 features in the FAS range (a thin upper lip, 4-Digit Code Lip Rank 4).

This results in almost every 4-Digit Code Facial ABC-Score meeting the relaxed Hoyme et al. facial criteria (Figure 5B) including 13 of the 15 ABC-Scores that depict the 4-Digit Code Rank 2 (mild) facial phenotype and 3 of the 8 ABC-Scores that depict the complete absence of all three FAS facial features (Rank 1). Clinically, the 4-Digit Code classifies Rank 1 and 2 facial phenotypes as being within the normal range. The practical clinical impact of this relaxation is illustrated in Figure 6 in which an adolescent with high function (e.g., FSIQ 123) and confirmed absence of prenatal alcohol exposure met the Hoyme et al. criteria for the full FAS facial phenotype.

In addition to the contrasts in facial criteria, the scales of measurement used to clinically classify the facial phenotype also differ. The 4-Digit Code documents the full continuum of expression of the FAS facial phenotype (Face Ranks 1 through 4), a continuum confirmed to be highly predictive of CNS dysfunction [5, 10]. In contrast, the Hoyme et al system documents the facial phenotype as simply present or absent.

##### CNS dysfunction:

The Hoyme et al. criteria that define neurobehavioral impairment (broadly defined as at least one neurobehavioral domain ≥1.5 standard deviations (SD) below the mean) appeared broadly equivalent to the 4-Digit Code criteria for moderate to severe CNS dysfunction (CNS Ranks 2 and 3). CNS Rank 3 (severe dysfunction, labeled Static Encephalopathy) is defined by 3 or more domains of function, 2 or more SDs below the mean. CNS Rank 2 (moderate dysfunction, labeled Neurobehavioral Disorder) is defined by at least one domain of function between 1 and 2 SDs below the mean, and not more than 3 domains of function 2 or more SDs below the mean. CNS Rank 1 reflects normal function across all domains [1]. Validation studies confirm CNS Ranks 1, 2 and 3 are significantly and linearly correlated with the severity of underlying CNS structural abnormalities, the magnitude of expression of the FAS facial phenotype, and the level of prenatal alcohol exposure [11].

##### CNS structural abnormalities:

The Hoyme et al. criteria for deficient brain growth, abnormal morphogenesis, or abnormal neurophysiology were largely equivalent to the 4-Digit Code criteria for CNS structural and neurological abnormalities (CNS Rank 4) with the exception of the cut-off used to define microcephaly (Hoyme et al. criteria: ≤ 10^th^ percentile; 4-Digit Code: ≤ 3^rd^ percentile).

##### Alcohol exposure:

The Hoyme et al., criteria for documented prenatal alcohol exposure are more stringent overall than the 4-Digit Code and include thresholds (≥6 drinks/week for ≥2 weeks during pregnancy or ≥3 drinks per occasion on ≥2 occasions during pregnancy). The 4-Digit Code requires a confirmed exposure, but does not set thresholds because recall and reporting of quantity, frequency, and timing of exposure have been confirmed highly unreliable in a clinical setting and exposure below a designated threshold has not been confirmed safe for all fetuses [11]. The Hoyme et al. system allows FAS and PFAS to be diagnosed when exposure is unknown. The 4-Digit Code allows FAS to be diagnosed when exposure is unknown because FAS requires the presence of the Rank 4 FAS facial phenotype and the Rank 4 face is confirmed to be highly specific to (caused only by) prenatal alcohol exposure [11].

## Objective 2: Comparison of diagnostic outcomes

### Methods

#### Study population

The records of 1,392 patients were drawn from 1,522 consecutive patients that received an FASD diagnostic evaluation at the University of Washington Fetal Alcohol Syndrome Diagnostic & Prevention Network (FASDPN). The diagnostic evaluations were performed by an interdisciplinary team between 1993 and 2012 using the FASD 4-Digit Code [1]. The interdisciplinary team included a medical doctor, psychologist, occupational therapist, speech language pathologist, social worker, family advocate, and public health professional [11, 12]. All patients with one or both birth parents African American (130 of the 1,522) were excluded from the study because it was unclear which PFL normal growth chart to use for African Americans when applying the Hoyme et al. system [13] and our findings in Objective 1 and those reported by Hoyme et al. [6] confirm the South African Mixed Race Lip/Philtrum Guide is inappropriate for use on an African American population.

Historically, all records resulting from each patient’s FASD diagnostic evaluation have been entered into a research database since 1992 with University of Washington Human Subjects approval and patient consent. Over 95% of patients provide consent for their clinical data to be used for research purposes. Patients’ records include the following standardized 4-Digit Code data forms: the New Patient Information Form, the FASD Diagnostic Form, digital facial photos, and the FAS Facial Photographic Analysis Report [1, 7]. These data are entered into a research database shortly after the patient’s FASD diagnostic evaluation reflecting the tools and growth norms available at that time. Over the decades the 4-Digit Code has evolved (First edition 1997, Third edition 2004) [1, 14–16], new tools have been developed like the FAS Facial Photographic Analysis Software (Version 1.0 in 2004, Version 2.1 in 2016) [7], and new more accurate growth norms have been adopted (CDC growth charts [17] and Stromland Scandinavian PFL charts [18].

For the purposes of research, all patients’ clinical 4-Digit Codes are updated to “research” 4-Digit Codes to reflect the most current tools and norms available at the time of the research study. For this study, all 4-Digit Codes were updated to reflect the most current third edition of the 4-Digit Code [1].

#### Application of the diagnostic tools and norms

The following tools and norms were used to update the 4-Digit Code FASD diagnoses and generate the Hoyme et al. [3] FASD diagnoses. The Reader is encouraged to familiarize themselves with the diagnostic criteria specific to each diagnostic system [1, 3] as space does not permit replication of the criteria here.

##### Growth:

The Hoyme et al. criteria use the same cutoff (prenatal or postnatal height and/or weight ≤ 10^th^ percentile) to define growth deficiency as the 4-Digit Code, thus all patients with 4-Digit Code Growth Ranks 2,3 or 4 were classified as meeting the Hoyme et al. growth deficiency criteria.

1. *Height and weight normal growth charts:* Height and weight percentiles were generated from the Hall [19] birth weight and length growth charts by gestational age; the World Health Organization (WHO) [20] height and weight growth charts for children 0–2 years of age, and the Centers for Disease Control (CDC) 2000 [17] height and weight growth charts for patients 2 years of age and older. The height percentile was adjusted for mid-parental height [21] when both parents’ heights were reported.

##### Facial features:

**Figure 7.**
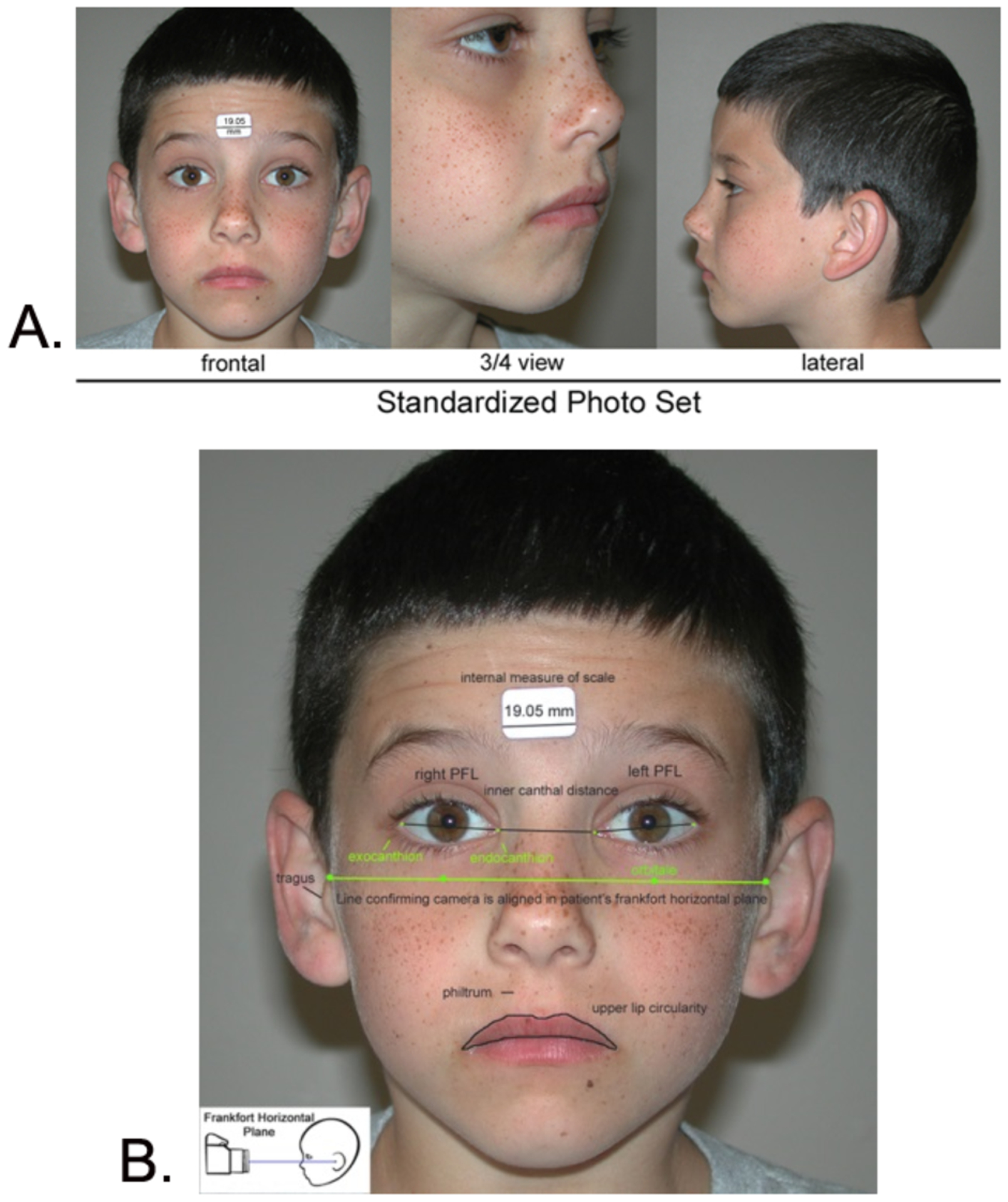
The FAS Facial Photographic Analysis Software was used to measure the 3 FAS facial features. A) The palpebral fissure length (PFL), philtrum smoothness, and upper lip thinness are measured from three standardized, digital photographs. B) Standardization includes proper rotation, exposure, focus, and facial expression. An internal measure of scale (a 3/4 inch (19.05 mm) paper sticker) is placed on the forehead to measure the PFLs in millimeters.

At the time of each patient’s FASD diagnostic evaluation, three standardized, digital facial photographs (Figure 7) were taken and measured using the FAS Facial Photographic Analysis Software [7]. As a result, each patient’s research record included the following facial measures: PFLs in millimeters, philtrum smoothness (Rank 1 to 5 on the 4-Digit Code Lip-Philtrum Guide 1) and upper lip circularity (perimeter^2^/area) and corresponding Lip Rank (Rank 1 to 5 on the 4-Digit Code Lip-Philtrum Guide 1).

1. *PFL:* For the purposes of this research study, all PFL z-scores were updated to reflect the Stromland Scandinavian PFL growth charts. The Stromland charts are confirmed valid for use on a North American population [8] and address the full age span (birth through adult) represented in our study population. In addition, the Stromland PFL growth charts were generated from digital images, thus meeting the recommendation by Hoyme et al. [3] that PFLs measured from photos should be compared to PFL normal growth charts generated from photos.
2. *Philtrum smoothness and upper lip thinness:* The 4-Digit Code “Caucasian” Lip-Philtrum Guide 1 and the Hoyme et al. North American Lip/Philtrum Guide were used to rank philtrum smoothness and lip thinness. Since the images depicting the Rank 1 through 5 philtrums on the 4-Digit Code and Hoyme et al. guides appeared broadly equivalent (per Objective 1), the philtrum rank assigned at the time of diagnosis using the 4-Digit Code guide was the same philtrum rank assigned to the patient using the Hoyme et al. guide (Figure 2) (e.g., if the patient had a Rank 4 philtrum using the 4-Digit Code guide, they received a Rank 4 philtrum using the Hoyme et al. guide). In contrast, the analyses in Objective 1 confirmed the Rank 1 through 5 images depicting upper lip thinness did not match between the 4-Digit Guide 1 and the Hoyme et al. North American Guide (Figure 2). The 4-Digit Code uses the full range of Lip Ranks 1–5 to classify the FAS facial phenotype on a 4-point Likert scale from normal (Face Rank 1) to severe FAS (Face Rank 4). In contrast, the Hoyme et al. FAS/PFAS facial criteria measure lip thinness on a dichotomous scale (thin: ≥Rank 4, not thin: <Rank 4) to classify the FAS/PFAS facial phenotype on a dichotomous scale (present, absent). To accurately and objectively identify which patients met the Hoyme et al. diagnostic criteria for a thin upper lip (≥Rank 4), the Rank 4 upper lip on the Hoyme et al. North American Lip/Philtrum Guide was outlined using the facial software. The video clip in Figure 2C demonstrates this procedure. The circularity of the Hoyme et al. Rank 4 lip was 52.5; equivalent to the 4-Digit Rank 2 lip (defined by the circularity range 42.5 to 57.4). Thus all patients with an upper lip circularity of 52.5 or greater met the Hoyme et al. criteria for a thin upper lip (Rank 4 or 5 on the Hoyme et al. North American Lip/Philtrum Guide).

##### CNS dysfunction:

Based on our findings in Objective 1, all patients with 4-Digit Code CNS Ranks of 2 or 3 (moderate to severe CNS dysfunction) were classified as meeting the Hoyme et al. criteria for neurobehavioral impairment.

##### CNS structural abnormalities:

Based on our findings in Objective 1, all patients with a 4-Digit Code CNS Rank 4 (structural/neurological abnormalities) were classified as meeting the Hoyme et al. criteria for deficient brain growth, abnormal morphogenesis, or abnormal neurophysiology. In addition, all patients with an occipital frontal circumference (OFC) ≤10^th^ percentile were classified as meeting the Hoyme et al. CNS criteria. The WHO [20] OFC charts for children 0–5 years of age and the Nellhaus [22] OFC growth charts for children 5–18 years of age were used.

##### Alcohol related birth defects (ARBD):

The Hoyme et al. diagnosis labeled ARBD is not recognized in the 4-Digit Code or any FASD diagnostic system introduced subsequent to the 1996 Institute of Medicine FASD diagnostic guidelines [12, 23]. For this reason, this diagnostic classification was not included in this study.

#### Statistical analyses

Descriptive statistics (valid percentages) were used to profile the study population. Chi-square tests were used to compare groups and linear trends across groups for outcomes measured on nominal or ordinal scales. One-way analysis of variance (ANOVA) was used to compare means and detect linear trends across three or more groups when outcomes were measured on a continuous scale. T-tests were used to compare means between two independent groups.

### Results

#### Objective 2: Compare the diagnostic outcomes between the two systems.

##### Study population

The socio-demographic profile of the study population (n=1,392) is presented in Table 1. The population spanned the entire age range from newborn to adult with 57% Caucasian and 44% female.

#### Objective 2a: Compare the prevalence of FASD diagnostic outcomes generated by each system

**Figure 8.**
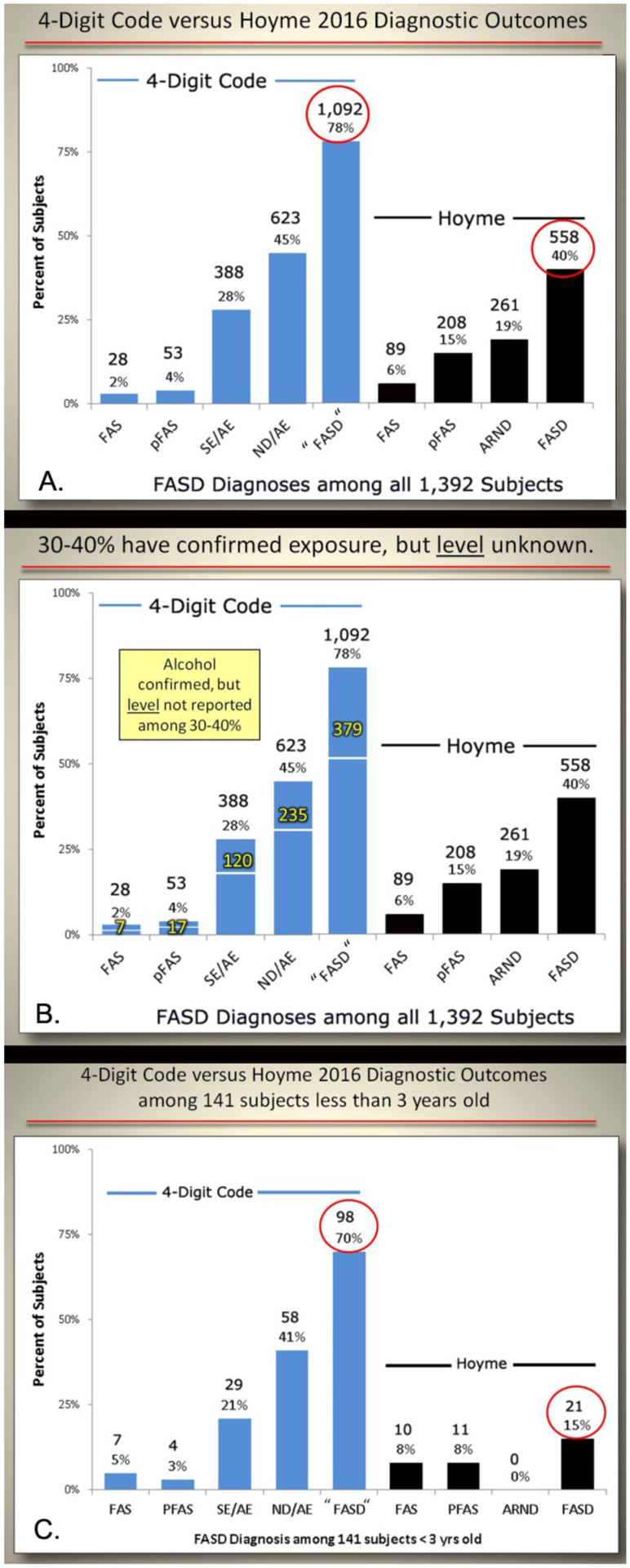
Contrasts in diagnostic outcomes when the 4-Digit Code and Hoyme FASD diagnostic systems were applied. A) Contrast in outcomes among all 1,392 patients. B) The impact of the more stringent Hoyme et al. [3] alcohol exposure criteria on the outcomes. The numbers in yellow document the number of patients with confirmed prenatal alcohol exposure that did not meet the more stringent Hoyme et al. criteria. For example, if the more stringent Hoyme et al. alcohol criteria were applied, 379 of the 1,092 would not have received a FASD diagnosis using the 4-Digit Code. C) Contrast in outcomes among the subset of 141 patients who were less than 3 years of age at the time of diagnosis. No infant/toddler received a diagnosis of ARND because the Hoyme et al. system does not permit a diagnosis of ARND in patients less than 3 years of age. FAS: fetal alcohol syndrome; pFAS: partial fetal alcohol syndrome; SE/AE: static encephalopathy/alcohol exposed; ND/AE: neurobehavioral disorder/alcohol exposed; FASD: fetal alcohol spectrum disorders; ARND: alcohol related birth defects.

The 4-Digit Code classified 78% (1,092/1,392) of the patients broadly under the umbrella of FASD (FAS, PFAS, SE/AE, and ND/AE) (Figure 8A). The prevalence of FAS and PFAS was 2% (n=28) and 4% (n=53), respectively. In contrast, the Hoyme et al. system classified only 40% (558/1,392) of the patients under the umbrella of FASD. The prevalence of FAS (6%; n=89) and PFAS (15%, n=208) generated by the Hoyme et al. system was 3-to 4-fold greater than the prevalence of FAS and PFAS generated by the 4-Digit Code.

**Figure 9.**
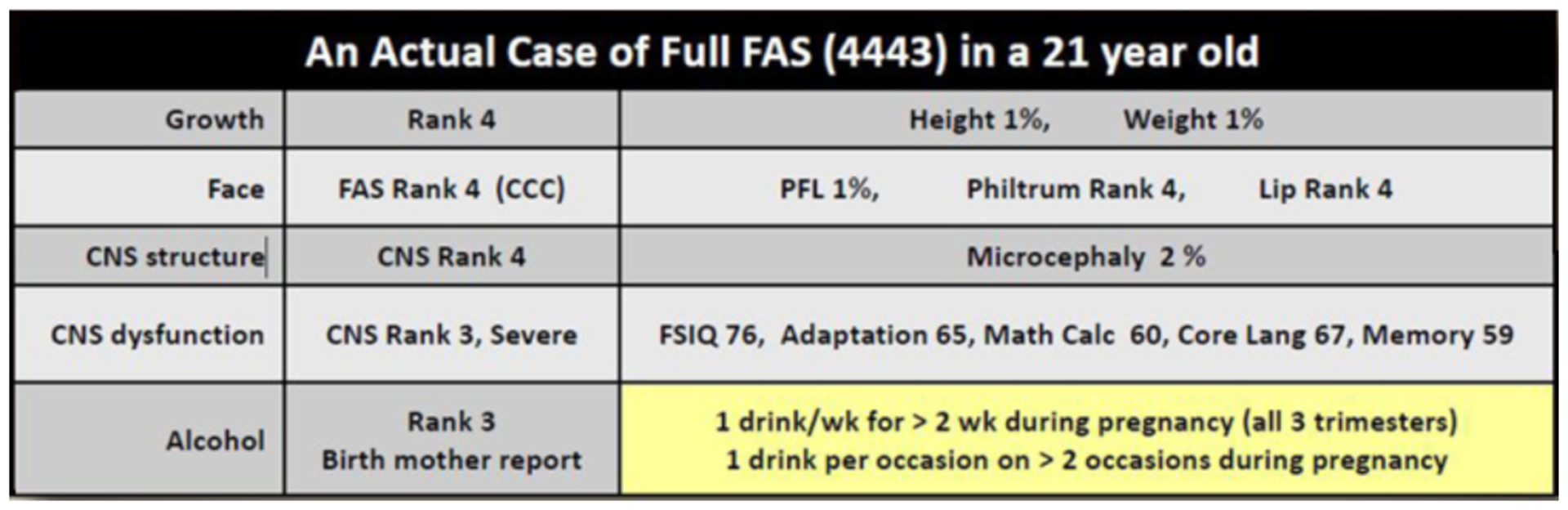
FAS does occur when the reported alcohol exposure is below the Hoyme et al. threshold. The outcomes displayed reflect an actual case of full FAS with confirmed prenatal alcohol exposure (4-Digit Code 4443) in a 21-year-old diagnosed at the University of Washington. This example demonstrates that FAS does occur when *reported* alcohol is less than the threshold (≥6 drinks/week for ≥2 weeks during pregnancy and/or ≥3 drinks per occasion on ≥2 occasions during pregnancy) required by the Hoyme et al. [3] FASD diagnostic system. The outcomes reported for CNS dysfunction are standard scores (mean 100, SD 15) for measures of cognition, adaptation, math calculation, core language skills, and memory. CNS: central nervous system; PFL: palpebral fissure length.

Thirty-five percent (379/1,092) of the patients who received a diagnosis of FASD using the 4-Digit Code did not receive a diagnosis of FASD using the Hoyme et al. system (Figure 8B). They all had confirmed prenatal alcohol exposures (e.g., birth mother reported drinking throughout the pregnancy), but their record of exposure did not meet the more stringent criteria (e.g., intoxication confirmed by BAC; positive biomarker test like analysis of FAEE; positive outcome on a validated screening tool like the T-ACE or AUDIT; or number of drinks per week or occasion reported) or the level of exposure (e.g., ≥ 6 drinks/week or ≥ 2 weeks or ≥2 drinks/occasion on ≥ 2 occasions) required by the Hoyme et al. system. In some cases, as illustrated in Figure 9, a patient diagnosed with severe FAS (4-Digit Code 4443) did not receive a diagnosis under the umbrella of FASD using the Hoyme et al. system because the exposure level reported directly by the birth mother (1 drink/week throughout pregnancy) was not high enough to meet the Hoyme et al. alcohol-exposure criteria (≥6 drinks/week for ≥2 weeks during pregnancy).

Among the subset of 141 alcohol-exposed patients under 3 years of age, the 4-Digit Code classified 70% (98/141) under the umbrella of FASD (Figure 8C). The prevalence of SE/AE and ND/AE was 21% (n=29) and 41% (n=58) respectively. In contrast, the Hoyme et al. system classified only 15% (21/141) under the umbrella of FASD. No infant/toddler received a diagnosis of ARND because the Hoyme et al. system does not permit a diagnosis of ARND in patients less than 3 years of age.

#### Objective 2b: Assess diagnostic concordance between the two systems

**Figure 10.**
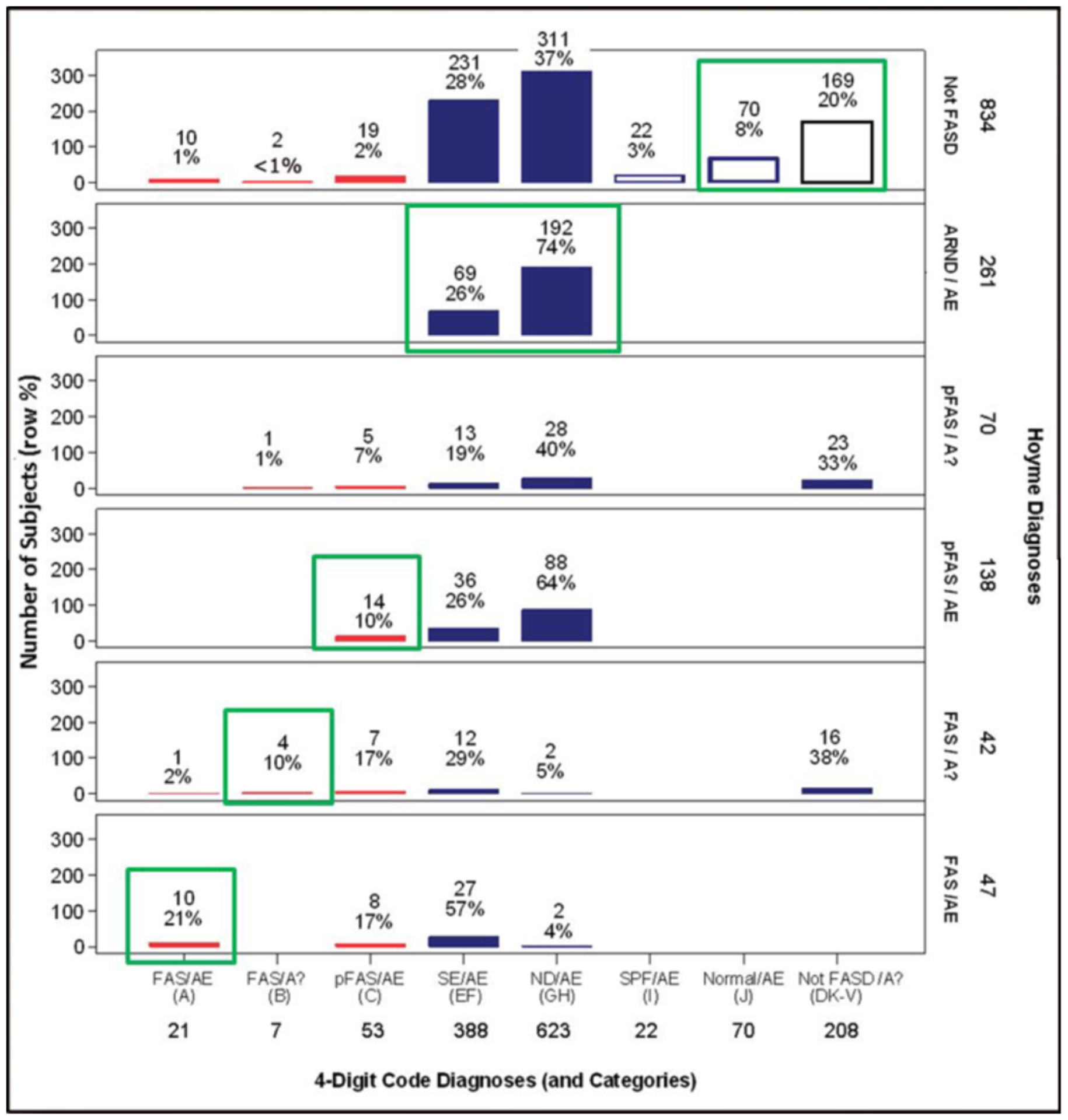
Cross-tabulation of the 4-Digit Code and Hoyme et al. FASD diagnostic outcomes. Diagnostic concordance (green boxes) between the two systems was observed in 38% (528/1,392) of the patients. Diagnostic discordance (all diagnoses not outlined in green) was observed in 62% (864/1,392) of the patients. Red bars reflect FAS and PFAS diagnoses using the 4-Digit Code. As a demonstration for how to interpret this figure; 21 patients received a 4-Digit Code Diagnosis of FAS/AE. Of the 21 patients, 10 received a FAS/AE diagnosis, 1 received a FAS/A?, and 10 did not receive a diagnosis under the umbrella using the Hoyme et al. [3] diagnostic system. 4-Digit Code Categories A-V are case-defined in the Diagnostic Guide for FASD [1]. AE: alcohol exposed; A?: alcohol exposure unknown; ND: neurodevelopmental disorder; Not FASD/A?: Individuals who present with or without growth, facial, and/or CNS abnormalitities, but are not under the umbrella of FASD because their prenatal alcohol exposure is unknown and they do not meet the criteria for FAS/A?. SE: static encephalopathy; SPF: Sentinel Physical Findings, individuals who present with growth deficiency and/or 1 to 3 FAS facial features, but have normal CNS structure and function; Normal: no evidence of growth, facial, or CNS structural/functional abnormalites.

Diagnostic concordance was observed in 38% (n=528) of the 1,392 patients (Figure 10). The two diagnostic systems ruled-out FASD in 239 patients and both rendered the same diagnosis under the umbrella of FASD for 289 patients. Diagnostic discordance was observed in 62% (n=864) of the 1,392 patients. The discordance ranged from subtle differences (e.g., the patient received a diagnosis of FAS by one system and PFAS by the other system) to marked contrasts (e.g., the patient received a diagnosis of FAS by one system and no diagnosis under the umbrella of FASD by the other system).

To illustrate some of the more striking contrasts, of the 21 patients that received a diagnosis of FAS/Alcohol Exposed using the 4-Digit Code, 10 had FASD ruled-out altogether using the Hoyme et al. system (see the 4-Digit Code FAS/AE column in Figure 10). All 10 patients were less than 5 years of age. They presented with CNS structural abnormalities (e.g., microcephaly: OFC ≤ 3^rd^ percentile), but early development was broadly within the normal range. All ten were too young to engage in the necessary level of testing to accurately rule-out moderate or severe CNS dysfunction. The Hoyme et al. system require both CNS structural abnormalities (e.g., OFC ≤ 10^th^ percentile) and evidence of CNS dysfunction for a diagnosis of FAS.

Among the 208 patients that were classified “Not FASD” by the 4-Digit Code, 39 received a FAS (n=16) or PFAS (n=23) diagnosis using the Hoyme et al. system (Figure 10). The 4-Digit Code does not render a diagnosis under the umbrella of FASD if: 1) alcohol exposure is unknown and 2) the Rank 4 FAS face is absent. If an individual does not have a confirmed prenatal alcohol exposure, the 4-Digit Code Rank 4 FAS face can serve as confirmation of exposure because the phenotype is confirmed to be so highly specific to (caused only by) prenatal alcohol exposure (>95% specificity) [11]. The Hoyme et al. system allowed these 39 patients with unknown alcohol exposures to receive a diagnosis of FAS or PFAS because they presented with the Hoyme et al. FAS face. But the Hoyme et al. FAS facial criteria are so relaxed (specificity 71% to 75% [24, 25]) that the facial phenotype does not provide the necessary level of specificity to alcohol to use the facial phenotype to confirm exposure. Among the 39 patients with unknown prenatal alcohol exposure and a Hoyme et al. diagnosis of FAS or PFAS, 18 had relaxed PFLs (4^th^-10^th^ percentile), 17 had relaxed philtrums (4-Digit Philtrum Ranks 2 and 3), 22 had relaxed lips (4-Digit Lip Ranks 1–3); 4 had no FAS facial features (4-Digit Face Rank 1); and 19 had only 1 FAS facial feature (4-Digit Face Rank 2).

Among the 834 patients that were classified “Not FASD” using the Hoyme et al. system, 31 received a FAS/PFAS diagnosis (Figure 10, red bars in the Hoyme et al. Not FASD row) using the 4-Digit Code. All 31 presented with the Hoyme et al. FAS face, but none met the Hoyme et al. FAS or PFAS diagnostic criteria. The Hoyme et al. FAS criteria require the presence of both CNS structural abnormalities (e.g., OFC ≤10th percentile) and neurobehavioral impairment. Eighteen presented with a small head circumference (OFC ≤10th percentile), but did not present with neurobehavioral impairment. All 18 were under 6 years of age. Of the 18 infants/toddlers, 5 were microcephalic (OFC ≤3rd percentile), but did not present with developmental delay ≥1.5 SD below the mean. All five were under 3 years of age and received the most severe FAS 4-Digit Code: 4444. Eight of the 31 presented with severe CNS dysfunction, but were normocephalic. Of the 29 with confirmed prenatal alcohol exposures, 5 had confirmed prenatal alcohol exposures, but the levels were reportedly too low to meet the Hoyme et al. alcohol exposure criteria.

**Figure 11.**
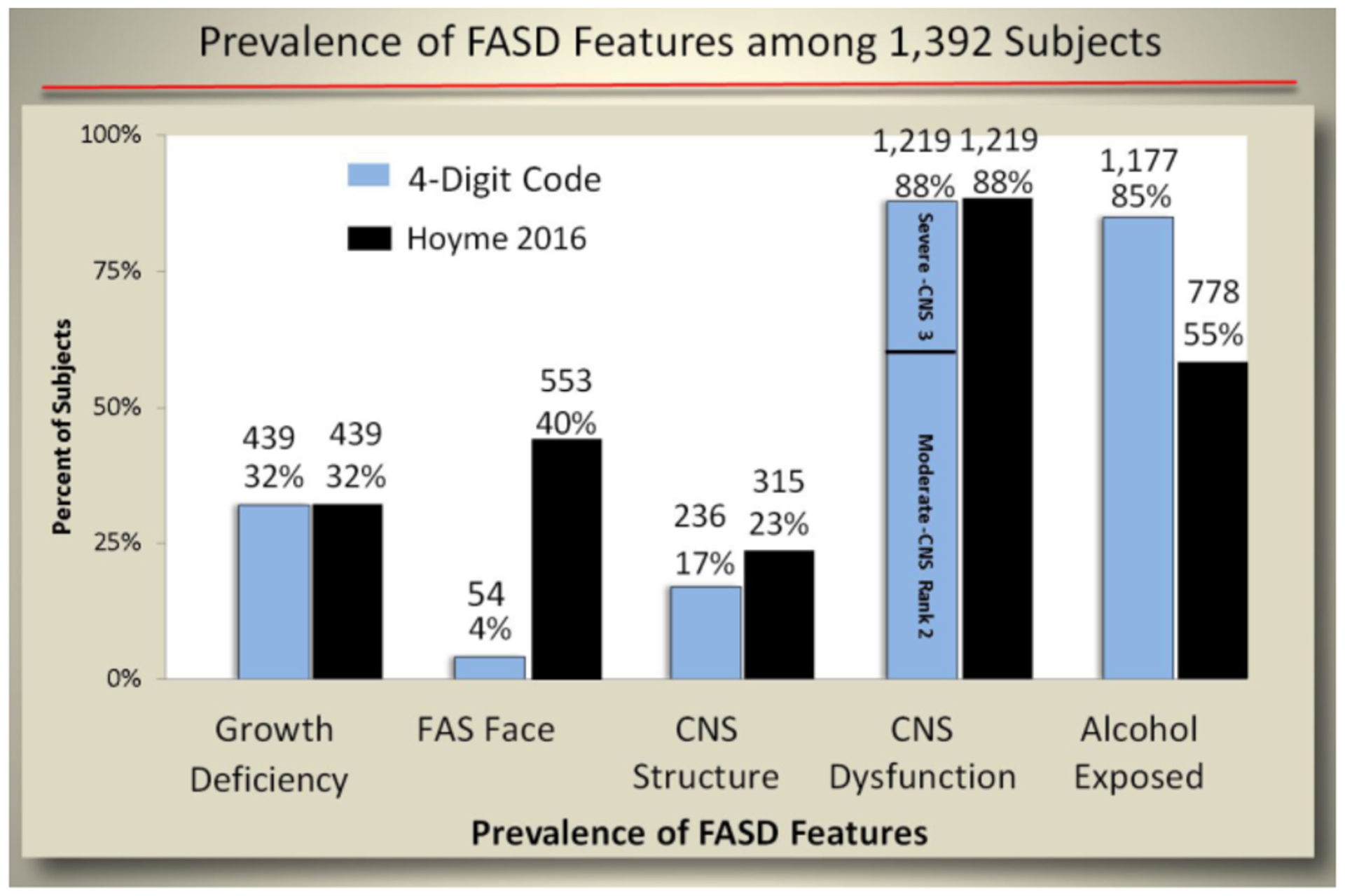
The prevalence of FASD features differed between the 4-Digit Code and Hoyme FASD diagnostic systems. The prevalence of growth deficiency, the FAS facial phenotype, CNS abnormalities, and alcohol exposure differed when the two diagnostic systems were applied to the 1,392 patients. The most striking contrast is the 10-fold higher prevalence of the FAS facial phenotype with the Hoyme et al. criteria. Of the 1,219 with CNS dysfunction, the 4-Digit Code identified 828 (68%) with Rank 2 moderate CNS dysfunction and 391 (32%) with Rank 3 severe CNS dysfunction. The blue bars reflect the 4-Digit Code: (growth deficiency=Growth Ranks 2–4; FAS face=Face Rank 4; CNS structure=CNS Rank 4; Alcohol Exposed=Alcohol Ranks 3 and 4). CNS: central nervous system.

The prevalence of each of the four core features that define FASD (growth deficiency, FAS facial phenotype, CNS abnormalities, and alcohol exposure) differed between the two diagnostic systems (Figure 11). Both systems identified 32% of patients with growth deficiency (height and/or weight ≤10^th^ percentile). The Hoyme et al. system identified a higher proportion of patients (23%) with CNS structural/neurological abnormalities than the 4-Digit Code (17%). The higher prevalence with the Hoyme et al. system reflected the relaxation of the OFC criterion to the 10^th^ percentile. Both systems identified 1,219 (88%) with CNS dysfunction. Of the 1,219 with CNS dysfunction, the 4-Digit Code identified 828 (68%) with Rank 2 moderate CNS dysfunction and 391 (32%) with Rank 3 severe CNS dysfunction. The prevalence of the FAS facial phenotype was 10-fold higher using the Hoyme et al. criteria (n=553; 40%) compared to the 4-Digit Code (n=54; 4%). Only 55% of the 1,392 patients met the more stringent Hoyme et al. alcohol exposure criteria. In contrast, 85% met the 4-Digit Code alcohol exposure criteria (Alcohol Rank 3 or 4).

**Figure 12.**
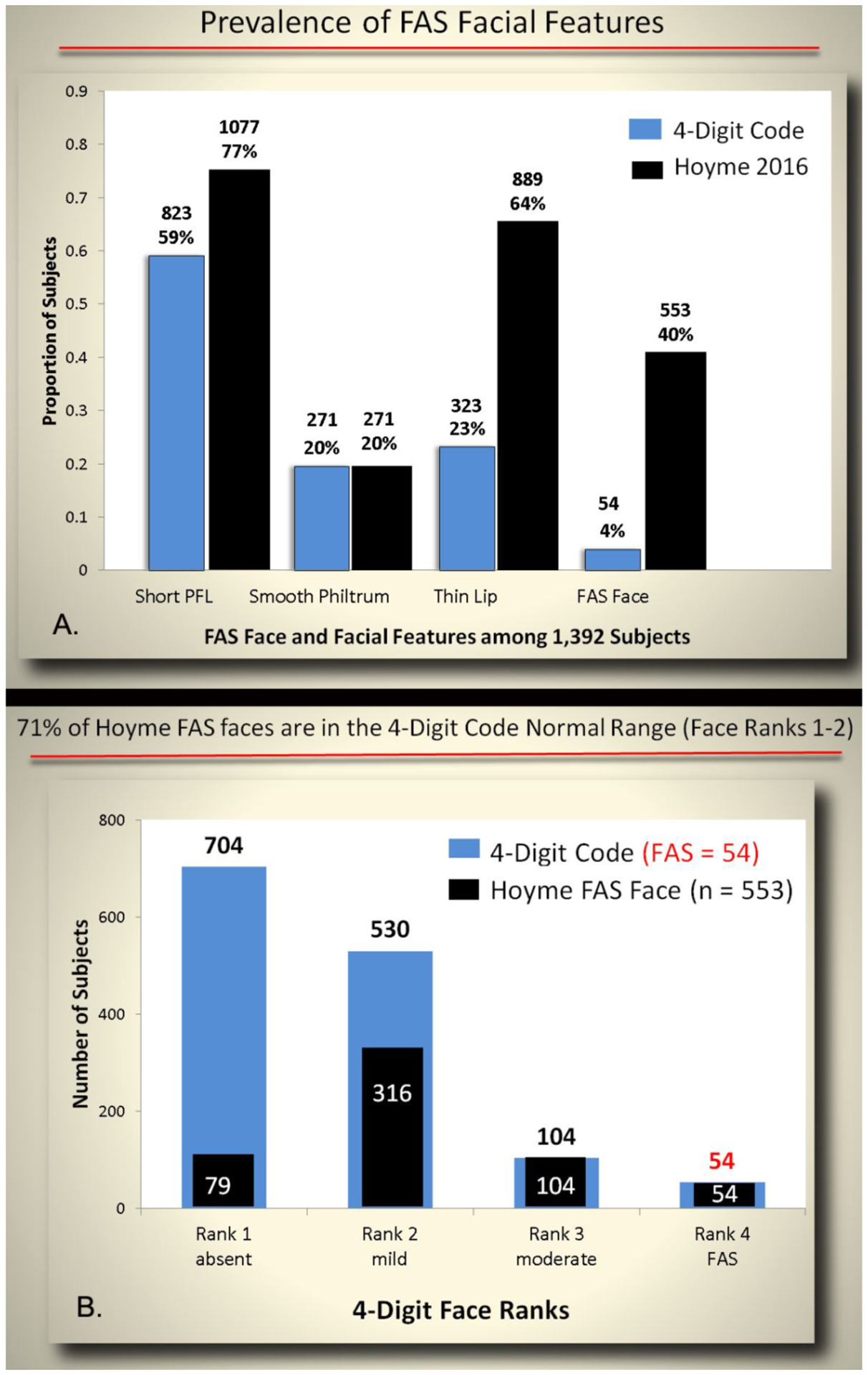
The prevalence of FAS facial features differed between the 4-Digit Code and Hoyme diagnostic systems. A) When the facial criteria from each diagnostic system were applied to the 1,392 patients, the most striking contrast was the prevalence of the thin upper lip. Use of the Hoyme et al. North American Lip/Philtrum Guide resulted in 3 times more patients being classified as having a thin upper lip. The criteria used to define each bar: Short PFL (4-Digit Code ≤3^rd^ percentile; ≤10^th^ percentile Hoyme et al.); Smooth Philtrum and thin upper lip (Rank 4 or 5 on the 4-Digit Code or Hoyme et al. [3] lip philtrum guides); FAS Face (4-Digit Code Face Rank 4; Hoyme al. FAS/PFAS face). B) 71% of the Hoyme et al. FAS facial phenotypes fell in the 4-Digit Code normal range (Face Ranks 1 and 2).

The prevalence of the individual FAS facial features also differed between the two diagnostic systems (Figure 12). More patients were classified with short PFLs using the Hoyme et al. system (77% ≤10^th^ percentile) than the 4-Digit Code (59% ≤3^rd^ percentile). Three times more patients were classified with thin upper lips using the Hoyme et al. Lip/Philtrum Guide (64% with Lip Rank ≥4; lip circularity ≥52.5) compared to using the 4-Digit Code Lip-Philtrum Guide (23% with Lip Rank ≥4; lip circularity ≥75.5). The philtrums depicted on the two lip-philtrum guides appeared to be roughly equivalent resulting in both diagnostic systems identifying 20% of patients with Rank 4 or 5 smooth philtrums. The relaxation of the PFL and lip criteria in addition to requiring only 2 of the 3 facial features resulted in the Hoyme et al. criteria identifying 10 times more patients with the full Hoyme et al. FAS face (n=553, 40%) than the 4-Digit Code FAS face (Rank 4; n=54, 4%). The relaxation of the Hoyme et al. FAS facial criteria resulted in 71% (395/553) of the Hoyme et al. FAS faces to fall within the clinically normal range (Face Ranks 1 and 2) as defined by the 4-Digit Code (Figure 12B).

**Figure 13.**
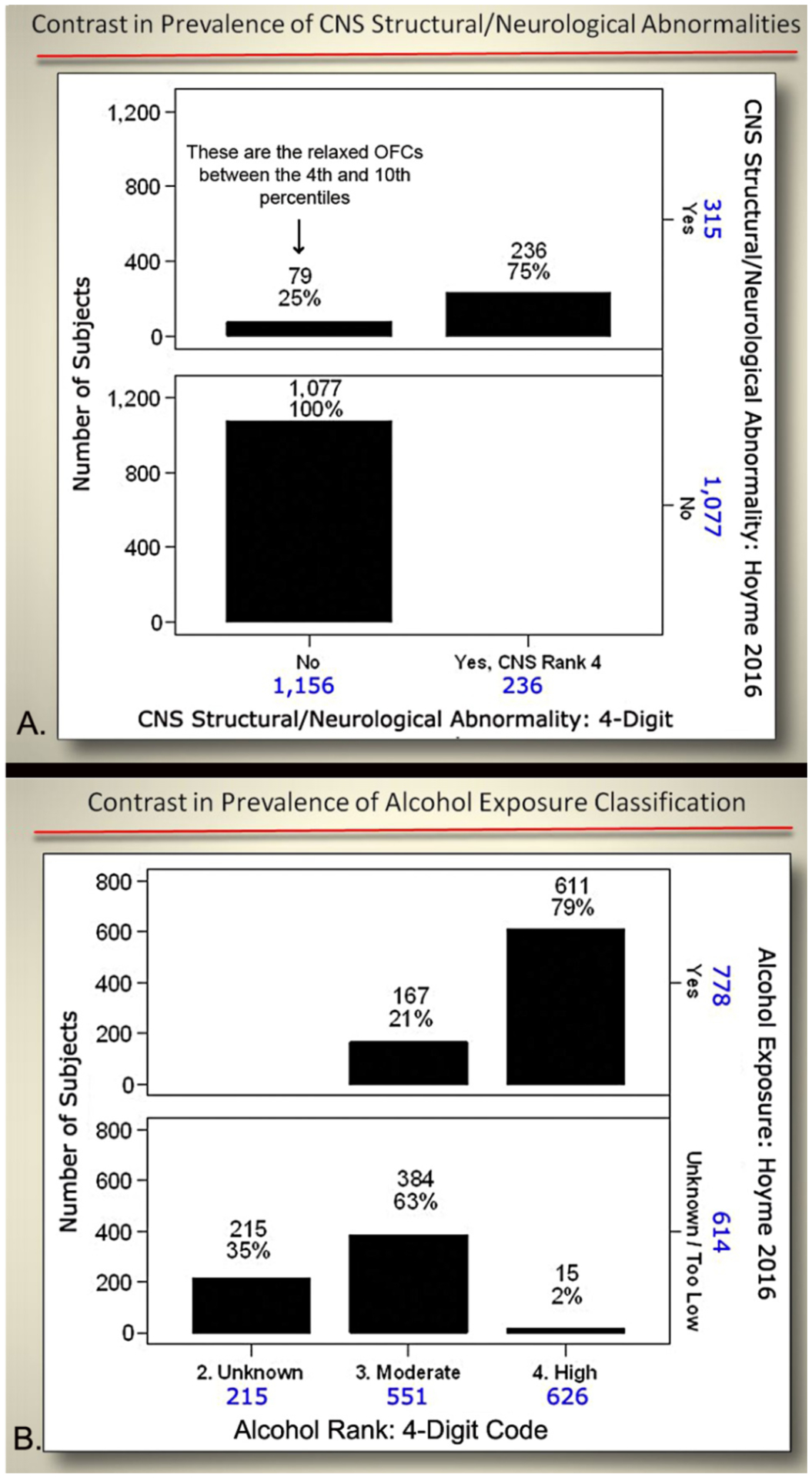
Cross-tabulation of CNS structural abnormalities and alcohol exposure classification between the 4-Digit Code and Hoyme systems. To aid in interpretation; A) 315 patients met the Hoyme criteria for CNS structural/neurological abnormalities. Seventy-nine of the 315 did not meet the 4-Digit Code criteria for CNS Rank 4 because the head circumference was between the 4^th^ and 10^th^ percentiles. The 4-Digit Code requires a head circumference ≦3^rd^ percentile. B) 552 patients were classified as having moderate prenatal alcohol exposure using the 4-Digit Code (Alcohol Rank 3). Of the 551, only 167 met the Hoyme criteria of alcohol exposure. The remaining 384 had confirmed exposures, but details on quantity, frequency, timing, blood alcohol levels, etc. were not available to meet the more stringent Hoyme criteria. OFC: occipital frontal circumference.

**Figure 14.**
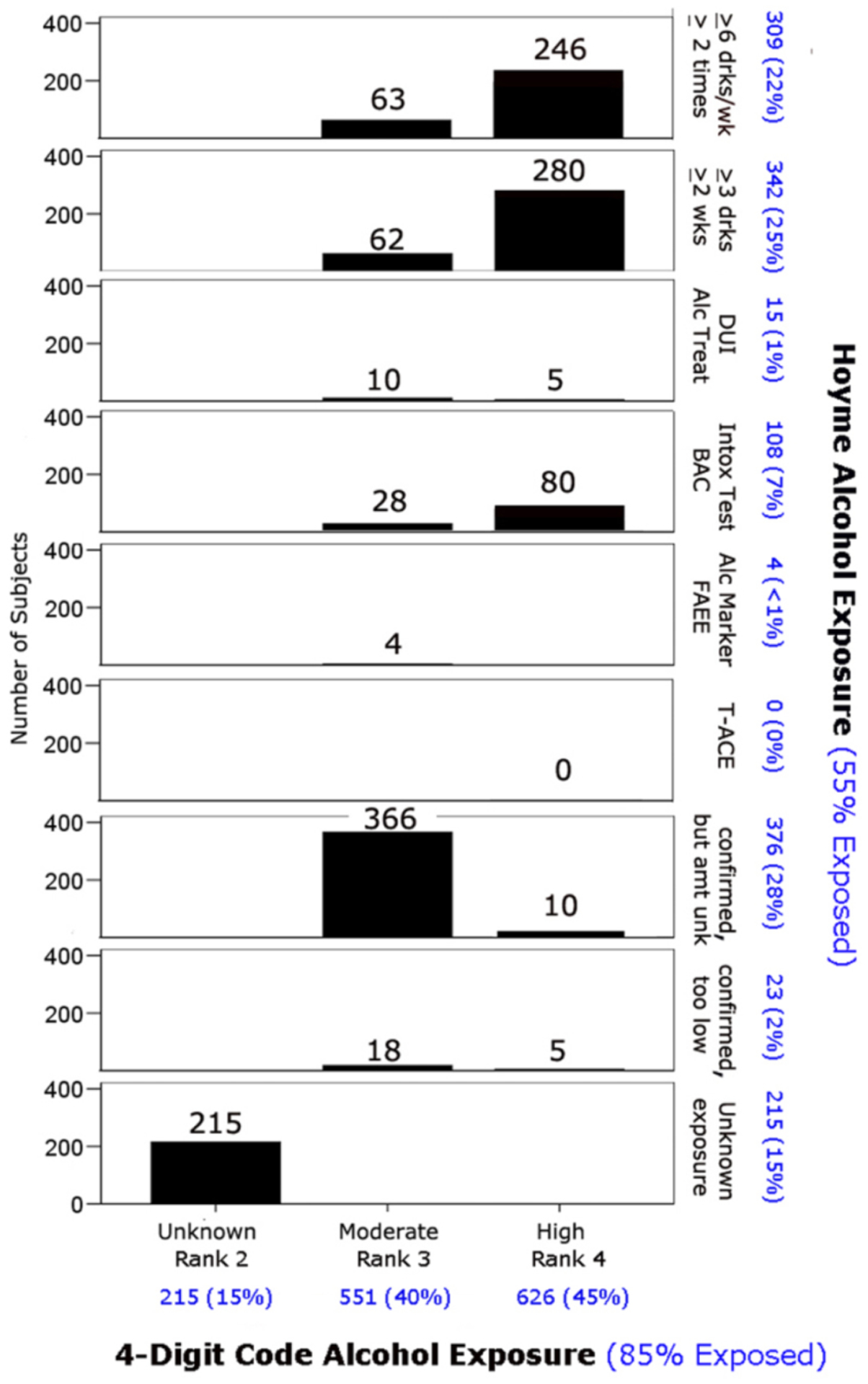
Cross-tabulation of the alcohol exposure classifications by the two 4-Digit Code and Hoyme diagnostic systems. The Hoyme et al. alcohol exposure categories listed along the right border are fully defined in Table 2 published in the Hoyme et al. diagnostic guidelines [3]. Patients in this study were classified into only one of these categories starting with the top category (e.g., if a patient was exposed to ≥6 drinks/week ≥2 times and had a DUI, they were classified only in the ≥6 drinks/week ≥2 times category). Overall, 55% of the patients met the Hoyme alcohol criteria, whereas 85% met the 4-Digit Code alcohol criteria.

Cross tabulation of the CNS structural abnormalities and alcohol exposure classification document further contrasts between the two systems (Figure 13). Relaxation of the head circumference criteria in the Hoyme et al. system resulted in 315 patients with OFC ≤10^th^ percentile compared to 236 ≤3^rd^ percentile using the 4-Digit Code. The more stringent Hoyme et al. criteria for alcohol exposure resulted in substantially fewer patients being classified as alcohol exposed (n=778, 55%) compared to the 4-Digit Code (n=1,177, 85%). Most notably, 399 patients with confirmed alcohol exposures (Rank 3 and 4) did not meet the more stringent Hoyme et al. alcohol criteria because the requisite details of exposure were unknown (e.g., quantity, frequency, timing, BAC, etc.). When the details of the Hoyme et al. alcohol criteria are displayed, it becomes more clear which of these criteria are most likely not to be met or available to clinicians (Figure 14).

#### Objective 2c: Assess measures of validation

##### Correlation between the FAS facial phenotype and prenatal alcohol exposure:

If the FAS facial phenotype is specific to (caused only by) prenatal alcohol exposure, the FAS facial phenotype should be more prevalent among those with higher exposure and should not occur in individuals with confirmed absence of prenatal alcohol exposure. One would also expect that the majority of (if not all) individuals presenting with the FAS facial phenotype would meet criteria for a diagnosis under the umbrella of FASD.

**Figure 15.**
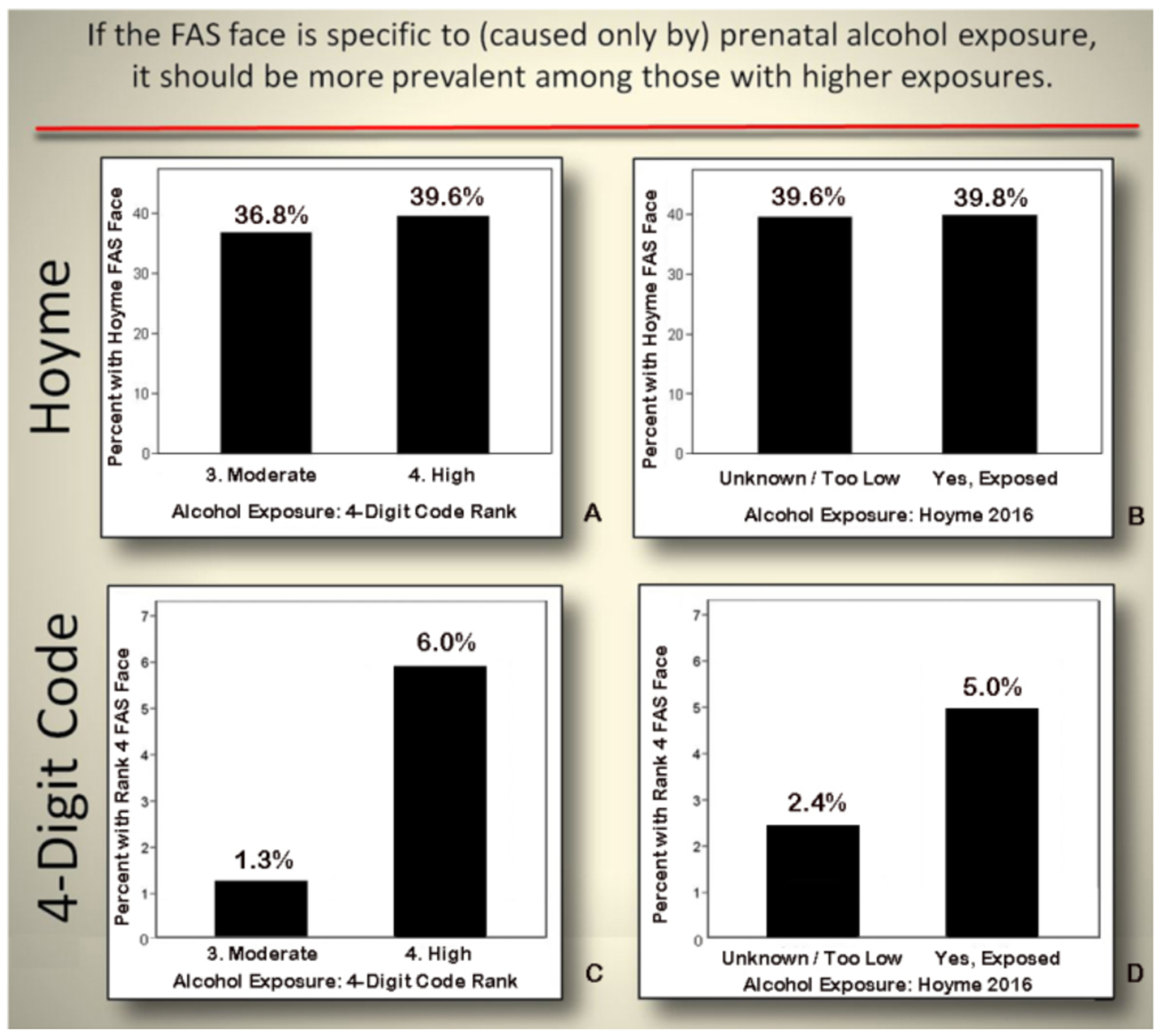
Only the 4-Digit Code FAS face was significantly more prevalent among patients with higher alcohol exposure. A) The Hoyme et al. FAS face was equally prevalent and highly prevalent in the moderate (4-Digit Code Alcohol Rank 3) and high (4-Digit Code Alcohol Rank 4) alcohol exposure groups (Chi^2^ 0.9, p=0.33). B) The Hoyme et al. FAS face was also equally prevalent and highly prevalent between those that did and did not meet the Hoyme et al. alcohol exposure criteria (Chi^2^ 0.01, p=0.92). In contrast, the 4-Digit Code FAS facial phenotype was highly correlated with measures of prenatal alcohol exposure. C) The 4-Digit Code Rank 4 FAS face was 5 times more prevalent in the high exposure group (4-Digit Code Alcohol Rank 4) than the moderate exposure (Digit Code Alcohol Rank 3) group Chi^2^ 17.5, p=.000). D) The association between the 4-Digit Code Rank 4 FAS facial phenotype and alcohol was substantially weakened when the Hoyme et al. criteria for alcohol exposure were applied (Chi^2^ 6.1, p=0.02). The 4-Digit FAS face was only 2-fold more prevalent in the Hoyme et al. exposed group relative to the Hoyme et al. unknown/too low exposure group.

**Figure 16.**
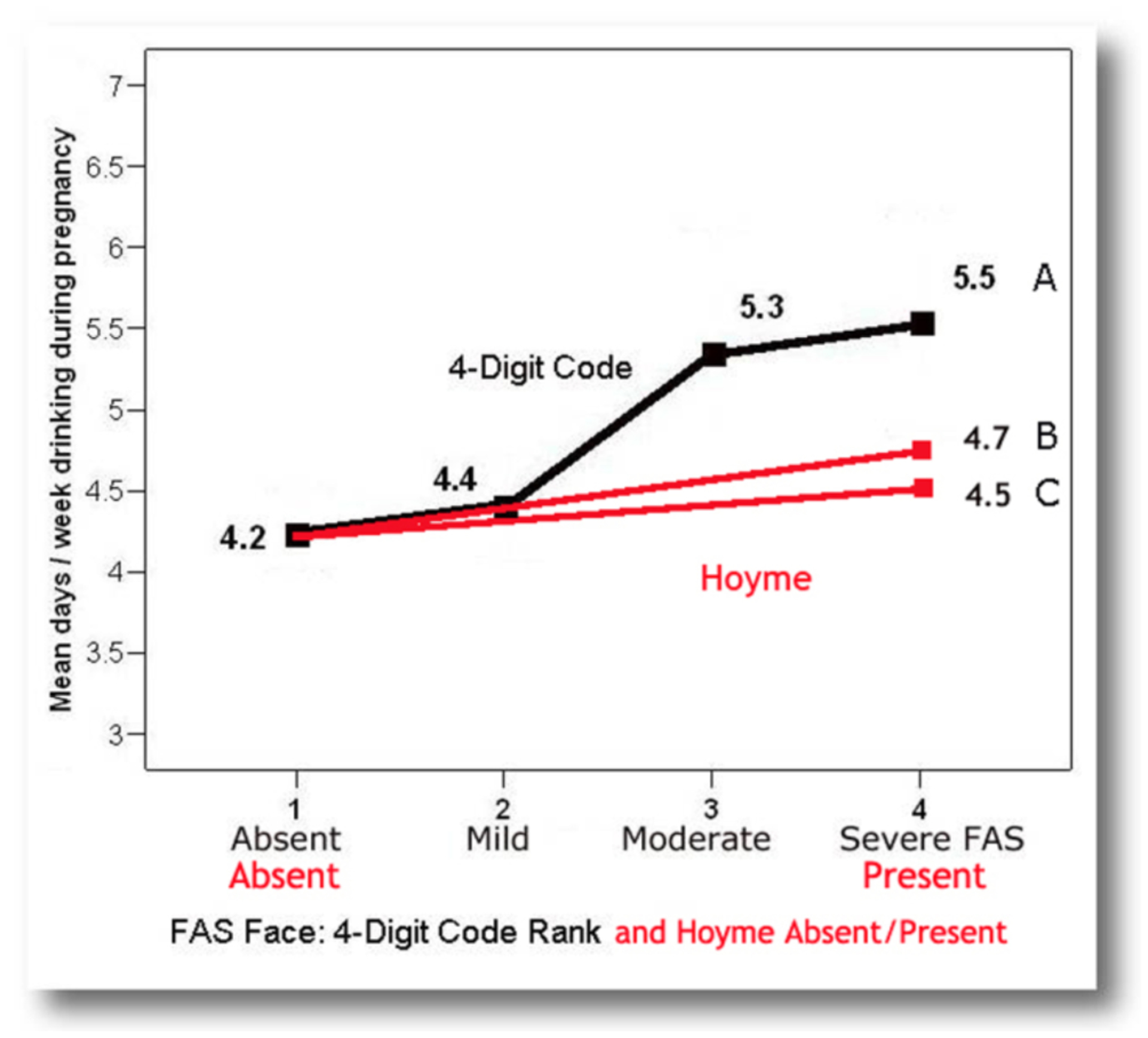
The 4-Digit FAS face was significantly associated with days/week of prenatal alcohol exposure. A) A strong, significant, linear association was observed between the mean number of days/week of drinking during pregnancy and increasing magnitude of expression of the 4-Digit Code FAS facial phenotype (Face Rank 1 to 4) (One-way ANOVA Linear term: F=12.7, p=.000; n=615). Patients with the full Rank 4 FAS facial phenotype were exposed, on average, 1.3 more days per week than the patients with no FAS facial features (Face Rank 1). B) A much weaker, but significant, association was observed between alcohol exposure and the Hoyme et al. [3] FAS facial phenotype. Patients with the Hoyme et al. FAS face were exposed, on average, 0.5 more days per week than the patients without the Hoyme et al. FAS face (4.7 days/week vs 4.2 days/week respectively; T=−2.9, p=0.04). Sixty-five of the 242 patients with the Hoyme et al. FAS facial phenotype had moderate to severe FAS facial phenotypes in accordance with the 4-Digit Code (Face Ranks 3 and 4). C) When these 65 patients were removed from the analysis to assess the correlation between the relaxed Hoyme et al. facial criteria and prenatal alcohol exposure, patients with the relaxed Hoyme et al. FAS face were exposed, on average, only 0.3 more days per week than the patients without the Hoyme et al. FAS face; a difference that was no longer statistically significant (4.5 days/week vs 4.2 days/week; T=−1.4, p=0.16).

The 4-Digit Code Rank 4 FAS facial phenotype was significantly more prevalent among patients with higher prenatal alcohol exposure (Figure 15C, D). In contrast, the prevalence of the Hoyme et al. FAS facial phenotype was not more prevalent among patients with higher alcohol exposures. (Figure 15A, B). The mean number of days/week of drinking during pregnancy increased significantly with increasing magnitude of expression of the 4-Digit Code FAS facial phenotype (Figure 16A). The mean number of days/week of drinking during pregnancy was only marginally higher among those with the Hoyme et al. FAS facial phenotype, but this was driven largely by the inclusion of 65 patients who also met the more stringent 4-Digit Code FAS facial criteria (Figure 16B). When these 65 patients were removed, there was no longer a significant contrast in alcohol exposure between those with and without the relaxed Hoyme FAS facial phenotype (Figure 16C).

When the Hoyme et al. and 4-Digit Code FAS facial criteria were applied to an adolescent with high function (FSIQ 123) and confirmed absence of prenatal alcohol exposure (4-Digit Code 1211), the adolescent met the Hoyme et al. criteria for the full FAS facial phenotype (Figure 6). In contrast, her facial phenotype was classified within the normal range by the 4-Digit Code (Face ABC-Score BBC, Face Rank 2).

Of the 553 patients with the Hoyme et al. FAS face, almost half (46%) did not receive a diagnosis under the umbrella of FASD using the Hoyme et al. system. In contrast, all 54 patients with the 4-Digit Code Rank 4 FAS face met criteria for a diagnosis under the umbrella of FASD using the 4-Digit Code.

## Discussion

The FASD 4-Digit Code and Hoyme et al. 2016 FASD diagnostic systems produced markedly different outcomes. Only 38% of the 1,392 patients received concordant diagnoses from the two systems. Overall, the Hoyme et al. criteria rendered half as many diagnoses under the umbrella of FASD (n=558) as the 4-Digit Code (n=1,092) and placed a much higher proportion (53%; 297/558) in the FAS/PFAS categories than the 4-Digit Code (7%; 81/1,092).

Four factors accounted for the greatest contrasts in diagnostic outcomes between the two systems.

1. **The more stringent Hoyme et al. alcohol exposure criteria prevented many with confirmed exposures from receiving a diagnosis of FASD**. These more stringent criteria prevented almost one third (339; 29%) of the 1,177 patients with confirmed exposure from being able to receive a diagnosis under the umbrella of FASD (Figure 14). As we illustrated in Figure 9, individuals with *reported* prenatal alcohol exposures below the Hoyme et al. threshold can and do present with full FAS when using the 4-Digit Code. Either this patient was particularly vulnerable to the teratogenic impact of alcohol, or the *reported* exposure was not accurate. In a clinical setting, one is never in a position to know how accurate the exposure was recalled and reported. Setting a threshold implies the details of all reported exposures are accurate and no fetus can be harmed by exposures below the threshold. Neither statement is true and the latter sends a dangerous public health message that lower levels are safe. Recognizing this, the 4-Digit Code requires a confirmed exposure, but does not set a threshold.
  It is interesting to note that Petryk et al., [26] reported similar findings when they retrospectively assessed the impact of applying the Canadian [2] minimal prenatal alcohol exposure thresholds to 119 patients with confirmed prenatal alcohol exposure (4-Digit Code Alcohol Ranks 3 or 4) and severe structural and/or functional CNS abnormalities (4-Digit Code CNS Ranks 3 and/or 4). The more stringent Canadian [2] exposure criteria would have prevented 71% of these individuals from receiving a diagnosis under the umbrella of FASD because the reported exposure would not have met the required threshold.
2. **The Hoyme et al. criteria require both CNS structural and functional abnormalities be present to receive a diagnosis of FAS**. Almost half of the patients who met the 4-Digit Code criteria for FAS did not meet the Hoyme et al. criteria for FAS because the patients were microcephalic, but too young (<5 years old) to engage in the types of testing needed to identify moderate or severe CNS dysfunction. The 4-Digit Code has confirmed that over 90% of alcohol-exposed infants and toddlers who present with one or more of the sentinel physical features of FAS as defined by the 4-Digit Code (microcephaly ≤3^rd^ percentile, a Rank 4 FAS facial phenotype, or Rank 4 growth deficiency) will present with severe CNS Rank 3 dysfunction later in childhood [5]. FAS is a birth defect syndrome, thus, by definition, it is present at birth. Failure to identify and diagnose FAS in newborns and infants will prevent these highest-risk children from receiving the benefits of early intervention. The 4-Digit Code allows evidence of CNS structural or functional abnormality to meet the CNS criteria. This allows FAS to be diagnosed in the newborn/infant who presents with the physical features (growth deficiency, the FAS facial features, and microcephaly), knowing these sentinel features are highly predictive of underlying CNS dysfunction that will manifest later in childhood.
3. **The Hoyme et al. criteria prevent children under 3 years of age from receiving a diagnosis of ARND**. As a result, 84% of the 87 alcohol-exposed infants/toddlers under 3 years of age that presented with moderate to severe CNS dysfunction and received a 4-Digit diagnosis of ND/AE or SE/AE (Figure 8C) did not receive a diagnosis anywhere under the umbrella of FASD using the Hoyme et al. system. Since ARND, by definition, is Neurodevelopmental Disorder caused by prenatal alcohol exposure, individuals with ARND are born with ARND. Failure to diagnose ARND in alcohol-exposed infants less than 3 years of age may prevent them from receiving the benefits of early intervention. The 4-Digit Code does not place age restrictions on any of the diagnoses under the umbrella of FASD.
4. **The relaxation of the Hoyme et al. FAS facial phenotype criteria greatly increased the prevalence of FAS and PFAS diagnoses and threatened the validity of these FAS and PFAS diagnoses**.
  - The Hoyme et al. system classified 10 times more patients with the FAS facial phenotype (n=553) than the 4-Digit Code (n=54).
  - The Hoyme et al. system produced 16 times more FAS/PFAS diagnoses with unknown alcohol exposure (n=112) than the 4-Digit Code (n=7). This is particularly concerning because 68 (61%) of these patients had 4-Digit Code Rank 1 or Rank 2 facial phenotypes that are, by our definition, clinically “normal”. The Rank 1 and 2 phenotypes have no specificity to prenatal alcohol exposure [27]. The only reason FASD diagnostic systems allow a diagnosis of FAS to be made when prenatal alcohol exposure is unknown is because the facial phenotype is so highly specific to (caused only by) prenatal alcohol exposure, the face serves to confirm the exposure. If the facial phenotype defined by the diagnostic system is not confirmed to be highly specific to alcohol, then: 1) the diagnosis cannot be validly labeled FAS or PFAS because a causal link cannot be confirmed between the patient’s alcohol exposure and their adverse outcomes, and 2) the facial phenotype cannot be validly used to confirm prenatal alcohol exposure when the history of exposure is unknown.
    - The 4-Digit Code allows a diagnosis of FAS to be made when prenatal alcohol exposure is unknown because the 4-Digit Code Rank 4 FAS facial phenotype is confirmed to be >95% specific to prenatal alcohol exposure [11, 28]. The 4-Digit Code does not allow a diagnosis of PFAS to be made when alcohol exposure is unknown, because the facial criteria for PFAS is relaxed to a Face Rank 3, resulting in a subtle reduction in specificity. To err on the conservative side, the 4-Digit Code requires a confirmed exposure for PFAS.
    - In the current study the relaxed Hoyme et al. FAS facial phenotype demonstrated no association with prenatal alcohol exposure. In contrast, the 4-Digit Code FAS facial phenotype demonstrated a strong, significant, linear association with prenatal alcohol exposure.
  - The Hoyme et al. system produced 4 times more FAS/PFAS diagnoses (297) overall than the 4-Digit Code (n=81).
  - 70% of the 297 Hoyme et al. FAS/PFAS cases had “normal” 4-Digit Code Face Ranks 1 or 2.
  - 46% of the 553 patients with the Hoyme et al. FAS face did not receive a diagnosis under the umbrella of FASD using the Hoyme et al. system. In contrast, all 54 patients with the 4-Digit Code Rank 4 FAS face met criteria for a diagnosis under the umbrella of FASD using the 4-Digit Code.

In addition to the contrasts in diagnostic criteria, the methods and tools used to measure the facial features are also markedly different. The authors of the Hoyme et al. system “*feel direct examinations of facial features are more practical in an office setting.”* The 4-Digit Code advises measuring the facial features from 2D digital photos using the FAS Facial Photographic Analysis Software [7]. Empirical studies have confirmed the superior accuracy of the photo versus direct method of facial measurement [8, 9]. Significant contrasts also exist between the 4-Digit Code Lip-Philtrum Guide 1 and the Hoyme et al. North American Lip/Philtrum Guide. As illustrated in Figure 2, although the Hoyme et al. North American Lip/Philtrum Guide looks similar in appearance to the 4 Digit Code Lip-Philtrum Guide 1, these are not interchangeable tools. The lips ranked 1 through 5 on the Hoyme et al. Guide do not match the lips ranked 1 through 5 on the 4-Digit Code Guide. The lips on the 4-Digit Code Guide become progressively thinner as Rank increases from 1 to 5. The lips on the Hoyme et al. guide do not become progressively thinner as Rank increases (e.g., the Hoyme et al. Rank 4 lip is thicker than the Hoyme et al. Rank 3 lip). The images used to depict lip thinness for each Rank do not match between the two guides. When the Hoyme et al. lips are mapped onto the 4-Digit Guide based on the objective measure of thinness (circularity), the Hoyme et al. Rank 1, 2, 3, 4, and 5 lips are equivalent to the 4-Digit Code Lip Ranks 2, 2, 3, 2, and 5, respectively. Both systems define the thin upper lip of FAS as Rank 4 or thinner. But the Hoyme et al. Rank 4 lip is substantially thicker than the 4-Digit Rank 4 lip (it is equivalent to the 4-Digit Rank 2 lip). The introduction of the Hoyme et al. North American Lip/Philtrum Guide serves to further relax the Hoyme et al. FAS facial phenotype. Only 2 of the 3 cardinal features are required and 2 of the 3 features are relaxed relative to the 4-Digit Code. The PFL is relaxed from the 3^rd^ percentile to the 10^th^ percentile and lip thinness is relaxed from Rank 4 to Rank 2 on the 4-Digit Code Lip-Philtrum Guide 1. An individual presenting with PFLs at the 10^th^ percentile, a Rank 1 deeply grooved philtrum, and a 4-Digit Code Rank 2 moderately thick upper lip would meet the Hoyme et al. criteria for the full FAS facial phenotype. The presence of a single, very minor anomaly (PFL at the 10^th^ percentile) does not constitute a dysmorphic facial phenotype. In fact, it would be difficult to justify classifying any of these three features as minor anomalies outside the normal range. Yet, this facial phenotype is used by the Hoyme et al. system to confirm prenatal alcohol exposure when prenatal alcohol exposure is unknown. Of the 71 patients with unknown prenatal alcohol exposure and the Hoyme et al. FAS facial phenotype, 70% had a 4-Digit Code Rank 1 or Rank 2 facial phenotype. By definition, 4-Digit Face Ranks 1 and 2 reflect normal phenotypes with no specificity to prenatal alcohol exposure. This was clearly illustrated in our FASD MRI study [27]. Sixteen high-functioning adolescents with confirmed absence of prenatal alcohol exposure were enrolled as controls in that study. Ten presented with Rank 1 facial phenotypes and 6 presented with Rank 2 facial phenotypes (one of which is illustrated in Figure 6). Based on the findings of the current study, the Hoyme et al. North American Lip/Philtrum Guide is not a valid tool for use with the 4-Digit Code. It is unfortunate and unclear why the Hoyme et al. system introduced a Lip-Philtrum Guide that emulates the 4-Digit Code Lip-Philtrum Guide (five images ranked 1 through 5 with Rank 4 identified as the clinical cut-off for the full FAS facial phenotype) when the Hoyme et al. FASD system only captures lip thinness and philtrum smoothness on a dichotomous (present/absent) scale. Only a single image (the Rank 4 image) is needed to classify the lip and philtrum as ≥Rank 4 on the Hoyme et al. North American Lip/Philtrum Guide. It is not clear why the tool includes Ranks 1, 2, 3 and 5 since none of these ranks or images are necessary to render Hoyme et al. FASD diagnoses.

**Figure 17.**
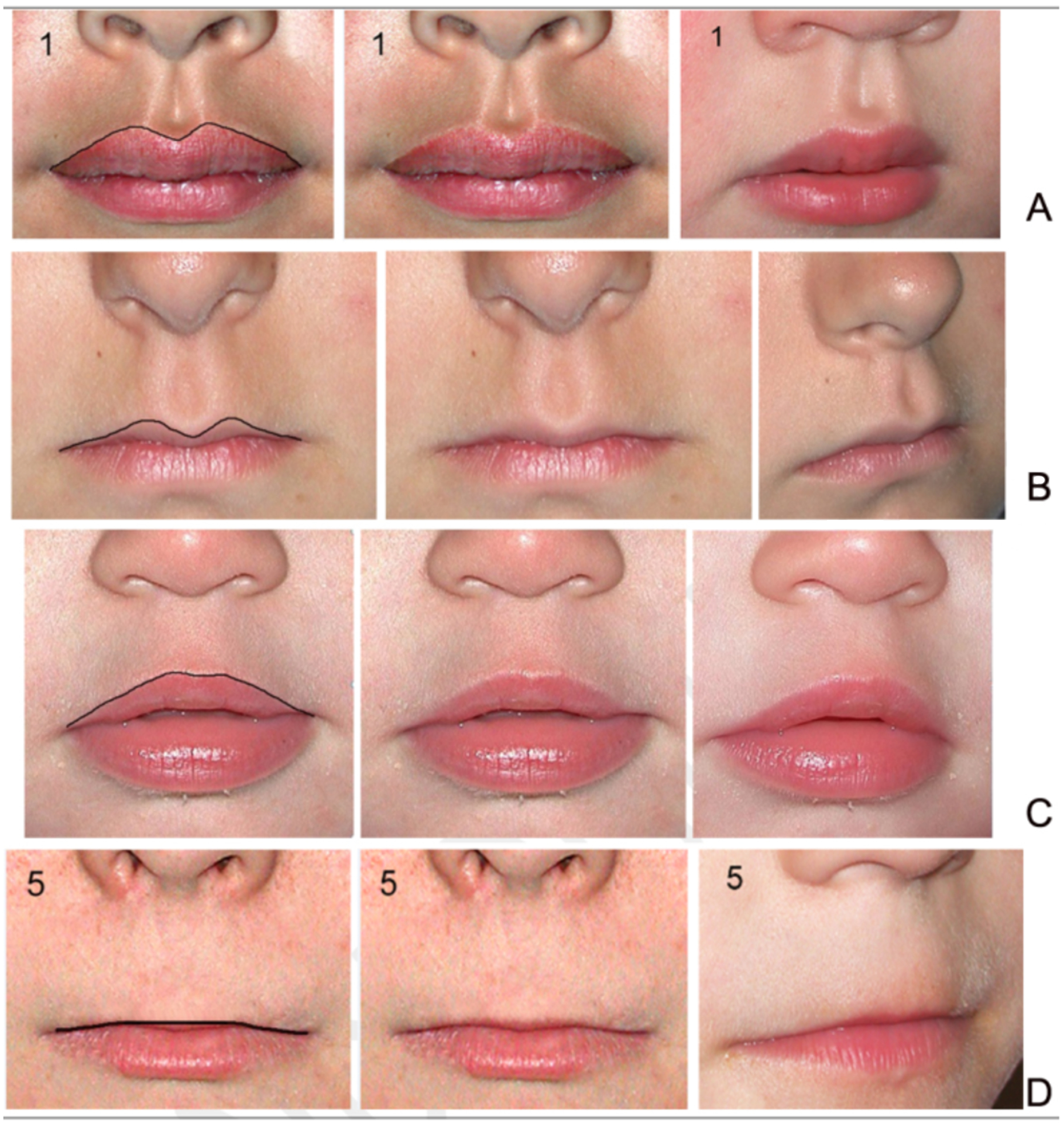
The absence of the Cupid’s bow is not a more precise method for documenting a thin upper lip The Cupid’s bow (black line) is the contour of the line formed by the vermilion border of the upper lip, resembling an archer’s bow in the frontal view. These images demonstrate that 1) the lower end of the philtrum groove and ridges form the Cupid’s bow [30], and 2) the absence of the Cupid’s bow is not a more precise method for documenting a thin upper lip [29]. The presence of a Cupid’s bow is dependent on the depth of the philtrum, not the thinness of the upper lip. A deep philtrum will form a Cupid’s bow even when the upper lip is thin. A and B) Examples of a deep philtrum creating a Cupid’s bow in the contour of a thick and thin upper lip. C and D) Examples of a smooth philtrum failing to create a Cupid’s bow in the contour of a thick and thin upper lip.

Before leaving the topic of the FAS facial features, the presence/absence of a Cupid’s bow warrants discussion. The Cupid’s bow is the contour of the line formed by the vermilion border of the upper lip, resembling an archer’s bow in the frontal view when a philtrum is present (Figure 17 A, B). Although the Hoyme et al.[3] diagnostic system clearly refers to the cardinal features of FAS as short palpebral fissures, smooth philtrum, and thin vermilion border of the upper lip and makes no reference to the Cupid’s bow, Del Campo and Jones [29] suggest the Cupid’s bow may be a more precise way to document a thin upper lip. Del Campo and Jones [29] describe a thin upper lip as follows, referencing the Hoyme et al. South African Mixed Race and North American White Lip/Philtrum Guides: “*A thin or narrow vermillion border of the upper lip has been considered another hallmark of the FAS since its initial definition. However many clinicians and investigators considered lack of the Cupid’s bow shape of the upper lip as more precise in order to evaluate this feature as shown for scores 4 and 5 of the lip/philtrum guide*.” The authors go on to report in the figure legend portraying the Lip/Philtrum Guides, “*For the vermillion border of the upper lip, the Cupid’s bow shape is either lost (*Rank *5) or very underdeveloped (*Rank *4).*” While some in the field have referred to a thin upper lip as a thin vermilion border, it is not the border that is thin, it is the vermilion (or red) part of the upper lip that is thin. This red portion of the upper lip is more accurately referred to as the upper lip vermilion.

It is interesting to note that the although lip thinness on the Hoyme et al. North American Lip/Philtrum Guide does not increase linearly with increasing Rank as one would expect, the contour of the Cupid’s bow does diminish linearly with increasing Lip Rank (Figure 2). Del Campo and Jones [29] appear to suggest that the lost or very underdeveloped Cupid’s bow shape of the upper lip may be a more precise way to evaluate the thin upper lip feature. We chose not to use the Hoyme et al. Lip/Philtrum Guide in this manner for the following reasons. First, the Hoyme et al. system makes no reference to the Cupid’s bow as a cardinal feature of FAS. Second, the lack of a Cupid’s bow reflects a smooth philtrum, not a thin upper lip. The Cupid’s bow is formed by the philtrum intersecting with the vermilion border of the upper lip, thus the Cupid’s bow is just another way of documenting the presence or absence of the philtrum (Figure 17). As defined by Hennekam et al. [30] in the Elements of Morphology, “*The philtrum is a vertical groove in the midline portion of the upper lip bordered by two lateral ridges or pillars It lies between the base of the nose (subnasale) and the vermilion border (labiale superius), which is also designated as the nasolabial distance. The lower end of the groove and the ridges form the central portion of the Cupid’s bow of the vermilion.”* And third, our dataset confirms that the absent or underdeveloped Cupid’s bow depicted as Ranks 4 and 5 on the Hoyme et al. Lip/Philtrum Guide are not exclusively associated with Rank 4 or 5 thin upper lips. Individuals with thin upper lips present with distinct Cupid’s bows when they have deep philtrums (Figure 17B) and individuals with thick upper lips have no Cupid’s bow when they have smooth philtrums (Figure 17C). As stated in the Elements of Morphology [31], “*A thin upper lip vermilion may be associated with a smooth philtrum and an absence of the Cupid’s bow, but these should be assessed separately.”* In the absence of published validation studies supporting this proposed change in one of the cardinal facial features of FAS, clinical teams should adhere to the thin upper lip vermilion feature that is thoroughly validated [11, 28].

One anticipated critique of our use of lip circularity in this analysis is that Hoyme et al. may intend for their lip-philtrum guide to be used for in-person visual comparison, not for photographic analysis using an objective measurement of lip thinness. We considered using retrospective visual comparison with clinic photographs using the Hoyme lip-philtrum guide, but determined that since the lips on the Hoyme guide do not become progressively thinner as Rank increases, and there is some confusion as to whether lip thinness or flat Cupid’s bow is being assessed with this guide, it would be too difficult to achieve adequate inter-rater reliability without relying upon the more objective measure of lip circularity.

**The quintessential role of the FAS facial phenotype**. Why are the criteria used to define the FAS facial phenotype so important to the medical validity of all diagnoses under the umbrella of FASD, not just the diagnosis of FAS? When one makes a diagnosis of FAS, one is stating explicitly that the individual has a syndrome caused by prenatal alcohol exposure [11]. One is also stating explicitly that the biological mother drank alcohol during pregnancy and, as a result, harmed her child. These are bold conclusions to draw and are not without medical, ethical, and even legal consequences. When the FAS face is not specific to FAS and prenatal alcohol exposure, the validity of the entire FASD diagnostic system collapses. Here is why.

- The term (FAS) is rendered invalid. If the face is NOT specific to (caused only by) alcohol, one can no longer label the condition fetal alcohol syndrome. One can no longer confirm alcohol is causally linked to any of the outcomes (growth, brain, or face) in an individual patient.
- The diagnosis (FAS with unknown alcohol exposure) is also rendered invalid. The FAS face can no longer serve as the confirmation of alcohol exposure when the exposure history is unknown.
- FAS is no longer distinct from ARND. ARND is essentially “FAS without the face.” But if there is no FAS face, there is no distinction between FAS and ARND. Thus, one can no longer justify classifying FAS and ARND separately.
- The term “ARND” remains problematic. Since ARND has no feature specific to prenatal alcohol, one is in no position to declare the Neurodevelopmental Disorder is “Alcohol-Related” (ARND) in an individual patient.

There are ethical consequences to the FASD diagnostic nomenclature. With a term like ARND, one feels compelled to require a significant exposure to alcohol to increase the odds that the individual’s impairments may be caused, at least in part, by their alcohol exposure. This is a dangerous road to go down.

- Setting a threshold of significant exposure for ARND does not confirm the patient’s alcohol exposure is related to their neurodevelopmental disorder.
- Alcohol is never the only risk factor contributing to the neurodevelopmental disorder. In this study population, 92% were exposed to other prenatal risks including poor prenatal care, pregnancy complications, and exposure to illicit drugs and tobacco. One percent presented with other syndromes (Down, Williams, Sticklers, etc.). Ninety-six percent experienced postnatal risks including trauma, neglect, multiple home placements, and physical/sexual abuse. Seventy-seven percent were in foster/adoptive care at the time of their FASD diagnosis. These other risk factors are so important in the differential diagnostic process, the 4-Digit Code Ranks the severity of these prenatal and postnatal factors on 4-point scales (just like it does for growth, face, CNS, and alcohol exposure) and reports them in full in the patient’s medical record.
- One is sending a dangerous message that lower levels of alcohol exposure are safe. As we illustrated in Figure 9, individuals with reported prenatal alcohol exposures below the Hoyme et al. threshold do present with full FAS. Either this patient was particularly vulnerable to the teratogenic insult of alcohol, or the reported exposure was not accurate. In a clinical setting, one is never in a position to know how accurate the exposure is recalled and reported. Setting a threshold implies the details of all reported exposures are accurate and no fetus can be harmed by exposures below the threshold.
- And one is blaming a woman for harming her child, when they have limited ability to make/defend such a claim.

The 4-Digit Code introduced the terms ND/AE and SE/AE back in 1997 [14]. These terms state the verifiable facts; the individual presents with a disorder and the individual was exposed to prenatal alcohol exposure. The terminology does not explicitly state their disorder is related to their alcohol exposure. In fact, the 4-Digit Code formally Ranks all other prenatal and postnatal risks factors to make clear that alcohol is never the only risk factor contributing to an individual’s neurobehavioral disorder of static encephalopathy. In 2013, the DSM5 [32] took a similar nosological approach when it introduced the new term “Neurobehavioral disorder associated with prenatal alcohol exposure” (ND/PAE) as a condition for further study.

### When is it a FASD?

Fetal Alcohol Spectrum Disorders are, by definition, adverse outcomes caused by prenatal alcohol exposure. In the absence of an outcome that is specific to (caused only by) prenatal alcohol exposure (like the Rank 4 FAS facial phenotype), one cannot confirm or rule-out the role prenatal alcohol exposure played in an individual’s CNS dysfunction. So…

- **Do all individuals with SE/AE, ND/AE (or ARND) have FASD?** Not necessarily. Only the subset of individuals whose CNS dysfunction was caused (in whole or in part) by their alcohol exposure has FASD.
- **Which subset is that?** We currently have no way of knowing. This is why the 4-Digit Code refers to SE/AE and ND/AE as ‘broadly” under the umbrella of FASD. Those with SE and ND caused by their alcohol exposure have FASD. Those with SE and ND that was not caused by their alcohol exposure do not have FASD.
- **But if they are exposed to high alcohol levels, can’t we just assume alcohol caused their disability?** No. Not everyone exposed to high levels of alcohol presents with adverse outcomes. Among 2,576 alcohol-exposed patients evaluated in the University of Washington FASDPN Clinic to date, 26 with high exposures presented with full FAS (4-Digit Codes 4444) while 41 with high exposures presented with normal growth, face, and brain development (4-Digit Codes 1114). We also see discordant outcomes among fraternal twins. Among 20 twin pairs with identical high exposures, 5 had normal CNS function while their twin had moderate to severe CNS dysfunction.
  When an individual presents with high alcohol exposure and severe CNS dysfunction, but no FAS facial phenotype, as depicted in the diagnosis SE/AE (4-Digit Code 2134):
  - If their CNS dysfunction is caused (at least in part) by their alcohol exposure, then their SE/AE is an FASD.
  - If their CNS dysfunction was caused by other risk factors, not their alcohol exposure, then their SE/AE is NOT an FASD.
  - The only way we can link alcohol to an individual’s CNS dysfunction is if they present with a highly specific Rank 4 FAS face (FAS 2434).
- **If we cannot confirm alcohol caused their disabilities, does this impact our ability to provide them appropriate intervention?** No. Intervention recommendations and a patient’s access to services and supports should be based on their disabilities, not on what caused their disabilities. Twenty years of patient surveys [33] confirmed patients with a diagnosis of ND/AE and SE/AE were as likely to access and benefit from interventions as patients with FAS/PFAS. We did not have to label their disorder FAS or PFAS to qualify them for intervention and support services in Washington State.
- **Does this impact our ability to prevent FASDs?** No. To prevent FASD one must prevent prenatal alcohol exposure. To confirm efforts to prevent prenatal alcohol exposure are working, one needs to document prenatal alcohol exposure in a patient’s medical record (regardless of outcome) and track the prevalence of prenatal alcohol exposure by birth cohort annually [34]. If one is reducing the prevalence of prenatal alcohol exposure, one is reducing the prevalence of FASD.

### Sensitivity versus specificity

Hoyme et al. (3) reported *“Sensitivity and specificity are 2 sides of a diagnostic coin. Theoretically, the guidelines presented here* (the Hoyme et al. 2016 guidelines [3]) *demonstrating increased sensitivity could lead to over-diagnosis; thus, our advocacy for a structured expert multidisciplinary approach. On the other hand, strict diagnostic cutoffs associated with increased specificity could lead to under-diagnosis of affected children. Children with FASD are subject to a host of societal, educational, health, and judicial problems, all of which are affected by the time of diagnosis. Because early diagnosis and initiation of intervention should be of paramount importance, the authors assert that improved, sensitive, and inclusive diagnostic criteria for FASD should continue to be imperatives in the diagnostic process.”* As demonstrated in the current study, strict diagnostic cutoffs (e.g., 3 facial features rather than 2, PFL 3^rd^ percentile rather than 10^th^ , lip Rank 4 rather than Rank 2; OFC 3^rd^ percentile rather than 10^th^) associated with increased specificity did not lead to under-diagnosis of affected children when using the 4-Digit Code. The 4-Digit Code uses stringent cutoffs for the FAS facial phenotype to achieve diagnostic accuracy/validity. If the face is not specific to (caused only by) alcohol, one cannot validly label the condition FAS (or PFAS) because one cannot link the patient’s adverse outcomes to their alcohol exposure. High specificity does not prevent individuals at risk for FASD from being identified and diagnosed. The 4-Digit Code is able to document the full continuum of outcomes and exposures (from 1113 to 4444) across the entire age span because it is not constrained by the implication of causation that comes with the term ARND. Aase and colleagues [35] urged “*simple recording of the verifiable conclusions. If prenatal alcohol exposure has taken place, but FAS cannot be substantiated, the exposure still should be indicated, and any nonspecific abnormalities or problems noted*.” This is the approach the FASD 4-Digit Code adopted when it was first introduced in 1997 [14]. This approach ensures no one is missed and no one is misdiagnosed.

**The two diagnostic systems produce different outcomes, but which one, if either, is correct?** Validation studies are required to confirm the accuracy, reproducibility, and medical validity of a diagnostic system. Validity is the degree to which a tool (or diagnostic system) is measuring what it purports to measure [36]. When the 4-Digit Code was introduced in 1997 [14, 16], it was published as an empirical study confirming its superior performance to the gestalt [37] approach it was designed to replace. Since then, two decades of more extensive laboratory, clinical, and public health empirical studies have comprehensively affirmed the validity of the FASD 4-Digit Code [11]. A clinician’s guide for how to fully assess the performance of FASD diagnostic systems was introduced in 2013 [11] and replicated below (Table 2). The guide proposes 12 questions clinicians should ask to assess the performance of FASD diagnostic systems. The 4-Digit Code’s performance meets all 12 criteria. In contrast, the 2005 [38] and 2016 [3] versions of the Hoyme et al. FASD diagnostic systems were introduced without empirical studies confirming its superior performance to other published FASD systems.

The single most important form of validation required of a FASD diagnostic system is confirmation that the FAS facial phenotype is highly specific to (caused only by) prenatal alcohol exposure. As discussed above, in the absence of a highly specific facial phenotype, the validity of the entire FASD diagnostic system collapses. The labels FAS and PFAS are rendered invalid. Diagnoses of FAS and PFAS can no longer be validly rendered when prenatal alcohol exposure is unknown. And FAS and PFAS are no longer distinguishable from ARND. Several published studies have confirmed that the Rank 4 FAS facial phenotype is highly specific (>95% specificity) [11] to prenatal alcohol exposure while the Hoyme et al. FAS Facial phenotype has a reported level of specificity (71% to 75%) [24, 25] that is insufficient to confirm it is caused only by prenatal alcohol exposure. This absence of association between the Hoyme et al. FAS facial phenotype and prenatal alcohol exposure was further confirmed in the current study. To date, the FASD 4-Digit Code is the only FASD diagnostic system that has a published record of validation.

## Conclusions

The FASD 4-Digit Code and Hoyme et al. 2016 FASD diagnostic systems produced markedly different outcomes. Only 38% of the 1,392 patients received concordant diagnoses from the two systems. Overall, the Hoyme et al. criteria rendered half as many diagnoses under the umbrella of FASD as the 4-Digit Code (558 and 1,092 respectively) due to more stringent alcohol exposure criteria and the setting of minimum age restrictions. The Hoyme et al. criteria placed a much higher proportion of FASD diagnoses in the FAS and PFAS categories than the 4-Digit Code (53% and 7%, respectively) because of the substantial relaxation of the Hoyme et al. FAS facial phenotype criteria. The Hoyme et al., FAS/PFAS facial phenotype had no correlation with or specificity to prenatal alcohol exposure. In contrast, the 4-Digit Code Rank 4 FAS facial phenotype was highly correlated with and highly specific to prenatal alcohol exposure. The Hoyme et al. North American Lip/Philtrum Guide and 4-Digit Code Lip-Philtrum Guide 1, while similar in appearance, are not equivalent tools. The Rank 4 moderately thin upper lip on the Hoyme et al. Guide is equivalent to the Rank 2 moderately thick upper lip on the 4-Digit Guide. The FASD 4-Digit Code has been extensively validated [11] over the past 20 years. In contrast, the relaxation of the FAS facial phenotype criteria poses a major threat to the validity of the Hoyme et al. 2016 FASD diagnostic system.

## Supplementary Material

Fig 2C video

## Figures and Tables

**Table 1.** Sociodemographic and exposure profile of the study population (n = 1,392)

Characteristic	N	Valid %
Gender		
Female	608	44%
Male	784	56%
Race/ethnicity		
Caucasian	788	57%
Native American	126	9%
Hispanic	37	3%
African American	0	0%
Other	434	31%
Age at FASD diagnostic evaluation (years)		
0–2	141	10%
3–5	314	23%
6–7	234	17%
8–12	411	30%
13–18	236	17%
19+	56	4%
Prenatal Alcohol Exposure		
Confirmed (4-Digit Code Ranks 3 and 4)	1,117	85%
Unknown (4-Digit Code Rank 2)	215	15%

**Table 2.** As clinicians assess the performance of FASD diagnostic guidelines, clinicians should ask the following questions [11]

Have properly designed studies been published to confirm the case definition for the FAS facial phenotype is highly specific (>95%) to FAS and alcohol (e.g. observed only among individuals with prenatal alcohol exposure and FAS)? If the FAS facial phenotype is not highly specific to prenatal alcohol exposure, FAS cannot be diagnosed when prenatal alcohol exposure is unknown Was data used to empirically derive the diagnostic guidelines? Was the data drawn from a large, representative, population-base? Has the performance of the guidelines been empirically assessed (validated)? Individuals are born with FAS/D. Can the diagnostic system identify FAS/D at birth and across the lifespan? Growth deficiency, the FAS facial phenotype, CNS abnormalities, and alcohol exposure all present along clinically meaningful continuums. The FAS facial phenotype is not just present or absent. The brain is not just normal or abnormal. Do the Guidelines recognize/incorporate these important continuums? Do the guidelines produce clinically distinct subgroups across the full spectrum (FAS, PFAS, SE/AE, ND/AE)? Do brain imaging studies identify statistically significant contrasts between the FASD subgroups? Individuals with FAS have more severe CNS dysfunction than individuals with “ARND”. Do the Guidelines generate FAS and “ARND” groups that demonstrate this important contrast? Do individuals who meet the criteria for FAS actually have FAS? Can the guidelines detect unique alcohol exposure patterns between the FASD subgroups? Can the diagnostic system be effectively and efficiently taught to interdisciplinary teams? Are the guidelines confirmed to be reproducible? If two clinics use the guidelines, do they render the same diagnoses? Do families report high satisfaction/confidence with the diagnostic process/outcome? Are the names of the diagnoses (FAS, PFAS, SE/AE, ND/AE) medically valid? Do they imply causality between alcohol and outcome that cannot be confirmed in the individual patient? Do diagnoses under the umbrella of FASD qualify patients for intervention services that lead to improved outcomes?
